# A gradual depth-dependent change in connectivity features of supragranular pyramidal cells in rat barrel cortex

**DOI:** 10.1007/s00429-014-0726-8

**Published:** 2014-02-26

**Authors:** Jochen F. Staiger, Ingo Bojak, Stéphanie Miceli, Dirk Schubert

**Affiliations:** 1 Institute for Neuroanatomy, University Medicine Göttingen, Kreuzbergring 36, 37075 Göttingen, Germany; 2 School of Systems Engineering, University of Reading, PO Box 225, Whiteknights, Reading, Berkshire RG6 6AY UK; 3 Donders Institute for Brain, Cognition and Behavior, Centre for Neuroscience, Radboud University Nijmegen Medical Centre, POB 9101//126, 6500 HB Nijmegen, The Netherlands

**Keywords:** Cortical microcircuits, Barrel-related column, Lemniscal system, Paralemniscal system, Caged glutamate

## Abstract

Recent experimental evidence suggests a finer genetic, structural and functional subdivision of the layers which form a cortical column. The classical layer II/III (LII/III) of rodent neocortex integrates ascending sensory information with contextual cortical information for behavioral read-out. We systematically investigated to which extent regular-spiking supragranular pyramidal neurons, located at different depths within the cortex, show different input–output connectivity patterns. Combining glutamate uncaging with whole-cell recordings and biocytin filling, we revealed a novel cellular organization of LII/III: (1) “Lower LII/III” pyramidal cells receive a very strong excitatory input from lemniscal LIV and much fewer inputs from paralemniscal LVa. They project to all layers of the home column, including a feedback projection to LIV, whereas transcolumnar projections are relatively sparse. (2) “Upper LII/III” pyramidal cells also receive their strongest input from LIV, but in addition, a very strong and dense excitatory input from LVa. They project extensively to LII/III as well as LVa and Vb of their home and neighboring columns. (3) “Middle LII/III” pyramidal cell shows an intermediate connectivity phenotype that stands in many ways in between the features described for lower versus upper LII/III. “Lower LII/III” intracolumnarly segregates and transcolumnarly integrates lemniscal information, whereas “upper LII/III” seems to integrate lemniscal with paralemniscal information. This suggests a fine-grained functional subdivision of the supragranular compartment containing multiple circuits without any obvious cytoarchitectonic, other structural or functional correlate of a laminar border in rodent barrel cortex.

## Introduction

The supragranular compartment within the primary somatosensory (barrel) cortex of rodents is difficult to separate into a genuine layer (L) II or III, due to its homogeneous appearance in cytoarchitectonic stains (Welker and Woolsey 1974). This has hampered progress in establishing cell type-specific and behaviorally relevant circuits with only few studies showing some aspect of LII versus LIII differences (Bureau et al. 2006; Shepherd and Svoboda 2005), whereas in most other studies neurons were pooled into a common LII/III.

Several forms of plasticity have been associated preferentially with LII/III (Bruno et al. 2009; Diamond et al. 1994; Feldman and Brecht 2005), suggesting that its synapses are highly mutable and constitute a substrate for learning and memory based on tactile information (Guic-Robles et al. 1992; Harris et al. 1999). Recent in vivo studies applying optogenetics, electrophysiology and calcium imaging have shown the behavioral relevance of these neurons, as well as their functional diversity (Chen et al. 2013; Feldmeyer et al. 2013; Houweling and Brecht 2008; Oberlaender et al. 2012; Petersen and Crochet 2013; Sato and Svoboda 2010). However, the precise underlying circuitry remains largely unclear.

In the barrel cortex, a whisker representation map is formed by neuronal clusters in LIV (Woolsey and van der Loos 1970). Spiny neurons within LIV issue strong and cell type-specific projections to the supragranular compartment (Staiger et al. 2004), where they preferentially form synapses with basal dendrites of pyramidal cells (Feldmeyer et al. 2002). This afferent input provides sensory information for integrative computations in LII/III (Waters et al. 2003), the outcome of which is then transferred to LVa/b (Adesnik and Scanziani 2010; Kampa et al. 2006). These projections together form an important part of the canonical microcircuit of cortical columns, which are the basic functional modules of the neocortex (cf. Douglas and Martin 2004; Feldmeyer 2012; Mountcastle 1997; Schubert et al. 2007). Nevertheless, neighboring supragranular excitatory neurons can differ considerably in their connectivity. These differences seem to depend on the functional circuit into which they are embedded rather than their particular (sub)laminar location (Benedetti et al. 2012; Brown and Hestrin 2009; Ko et al. 2011; Yoshimura et al. 2005).

To define the underlying differences in connectivity pattern of LII/III pyramidal neurons and to identify a possible laminar border within the LII/III, we performed a detailed mapping of monosynaptic, intracortical inputs onto pyramidal cells within the supragranular compartment of barrel-related columns using flash-release of glutamate and axonal reconstruction following whole-cell recording and biocytin filling. All recorded supragranular pyramidal cells showed a weak input from intralaminar sources. Our results define, for the first time, a gradual change in the source of input along the radial axis of LII/III, where pyramidal cells located in lower LII/III mainly received excitatory inputs from LIV (i.e., lemniscal) with a gradual transition of inputs targeting the upper LII/III, which are mainly arising from LIV but also prominently from LVa (i.e., paralemniscal; Bureau et al. 2006; Diamond et al. 2008; Wimmer et al. 2010; Yu et al. 2006). Analysis of the axon distribution suggested a stronger segregation of projections to the home column (including LIV) for lower LII/III. When approaching upper LII/III, a continuous change of the axon configuration was observed, with increased transcolumnar projections, necessary for effective cross-whisker integration of pyramidal cells within the supragranular layers of the barrel cortex. An important question for future studies will be how these circuits analyzed in vitro here, relate to the processing of sensory information in the behaving animal, where most in vivo studies have found sparse coding to be the typical scheme in supragranular layers II/III (Brecht et al. 2003; De Kock et al. 2007; De Kock and Sakmann 2009).

## Materials and methods

### Slice preparation and chemicals

All experiments were performed in accordance to the German and Dutch Law on the Protection of Animals. Male Wistar rats (postnatal days 20–25) were deeply anesthetized with isoflurane and decapitated. Brain slices of 300 μm thickness, containing the primary somatosensory cortex (barrel cortex; Paxinos and Watson 1998) were produced by sectioning either coronally following standard procedures or, for reasons of comparison with studies of other groups (Shepherd et al. 2003; Shepherd and Svoboda 2005), in an oblique angle approximately in parallel to the barrel arcs (Finnerty et al. 1999, VT1000S vibratome, Leica; Germany).

Slices were pre-incubated for 1 h at 34 °C and later kept at room temperature in oxygenated (carbogen 95 % O_2_/5 % CO_2_) artificial cerebrospinal fluid that was modified for cutting and storage purposes (cACSF). Compared to standard ACSF (in mM: 124 NaCl, 1.25 NaH_2_PO_4_, 26 NaHCO_3_, 1.6 CaCl_2_, 1.8 MgCl_2_, 3 KCl, 10 glucose, at pH 7.4), in cACSF the concentrations of Ca^2+^ and Mg^2+^ were modified to reduce neuronal activity (1 CaCl_2_, 4 MgCl_2_).

### Electrophysiology

**Fig. 1:**
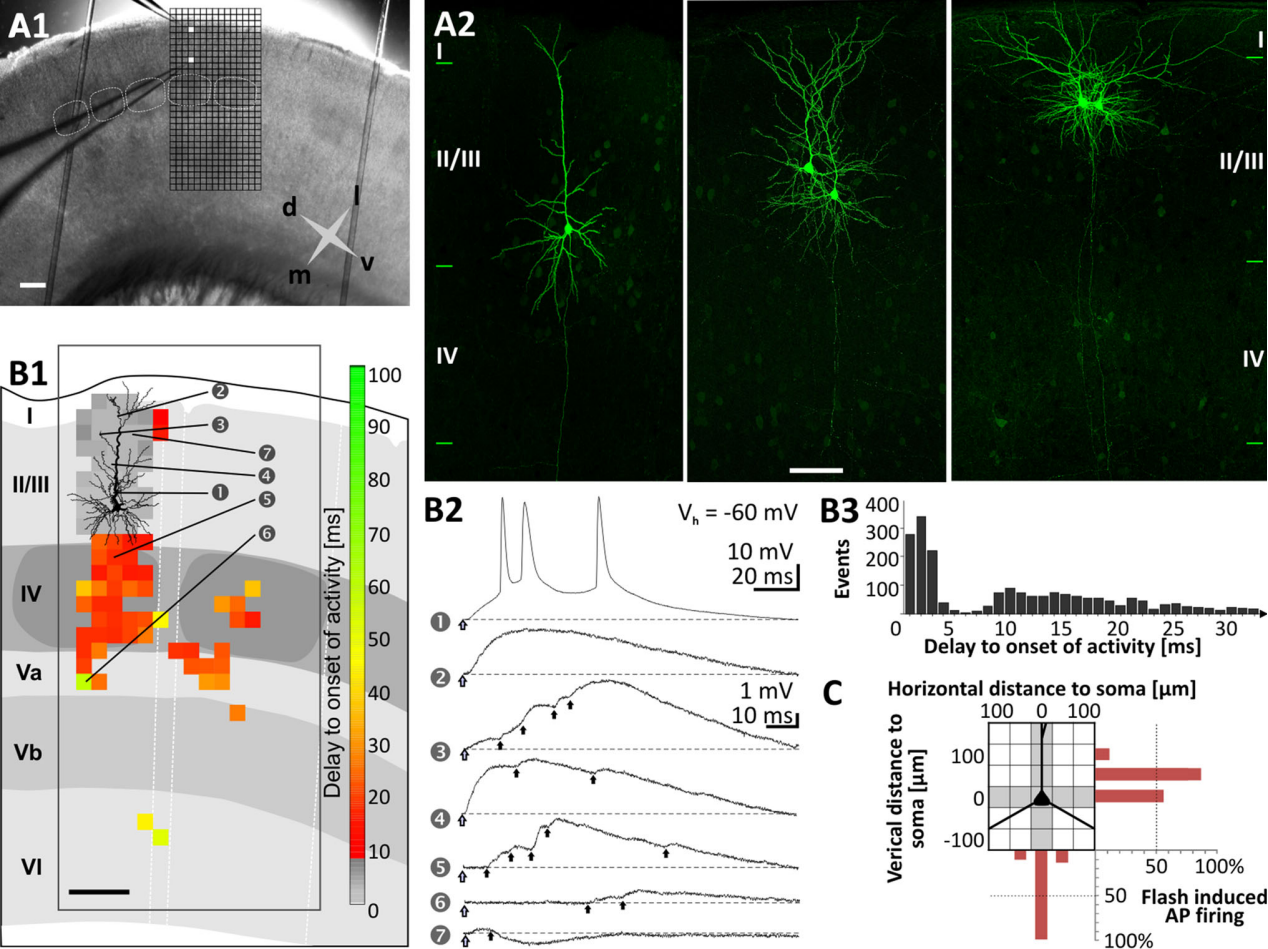
Combination of dual whole-cell patch clamp recording of supragranular pyramidal neurons and caged glutamate photolysis. **a1** Photomicrograph of a coronal slice of the rat primary somatosensory (barrel) cortex taken directly after an experiment with both recording electrodes positioned in the supragranular compartment. *White boxes* within the grid mark the position of the recorded pyramidal neuron somata in vertical alignment with a LIV barrel (*stippled white outlines*). At 10 s intervals, up to 450 fields of 50 × 50 μm in size (*black grid*) were stimulated in sequence covering all cortical layers and at least 2 barrel-related columns. The *cross* indicates medial (*m*), lateral (*l*), dorsal (*d*) and ventral (*v*) directions. **a2** Confocal images of biocytin-filled supragranular pyramidal cells after streptavidin-Alexa 488 staining. Cells were recorded (from *left* to *right*) in lower, middle and upper LII/III. **b** Direct responses and synaptically mediated activity in a LII/III pyramidal cell, induced by sequential uncaging of glutamate. **b1** Schematic illustration of the laminar and columnar organization of the cortical region investigated with caged glutamate photolysis. Superimposed are the somatodendritic reconstruction of the recorded neuron and a topographic map of origins of glutamate-induced activity. *Color code* represents the delay between flash stimulus and the onset of first detected flash-related activity in the recorded cell. Activity with delay to onset times <6 ms is restricted to fields containing dendrites of the recorded neuron and represents direct responses. **b2** Recordings of the membrane potential at *V*_h_ = −60 mV obtained after flash stimulation (*yellow arrows*) of fields as indicated by the numbers in **b1**. **b3** Histogram of the delays to onset of activity for all flash-induced responses of 35 completely mapped supragranular pyramidal neurons shows a clear separation of short latency direct responses from longer-latency synaptic responses. Direct responses, starting almost immediately after flash stimulation at perisomatic sites and reaching threshold in (*1*). Direct responses followed by flash-induced excitatory postsynaptic potentials (EPSPs, *3*, *4*). Flash-induced multiple EPSPs (*5*, *6*). Flash-induced inhibitory postsynaptic potential (IPSP); the IPSP truncates the weak preceding direct response (*7*). **c** Flash stimulation-induced action potential firing of supragranular pyramidal cells recorded at *V*_rmp_ (*n* = 41). Each square in the grid represents one stimulated field. Percentages represent the proportion of neurons where stimulating at a given horizontal or vertical distance to the recorded soma resulted in action potential (AP) firing. Note that there was no single case of AP induction below the soma. *Roman numerals* in all *Figures* denote cortical layers. *Scale bars* 200 μm (**a1**, **b1**); 100 μm (**a2**)

**Fig. 2:**
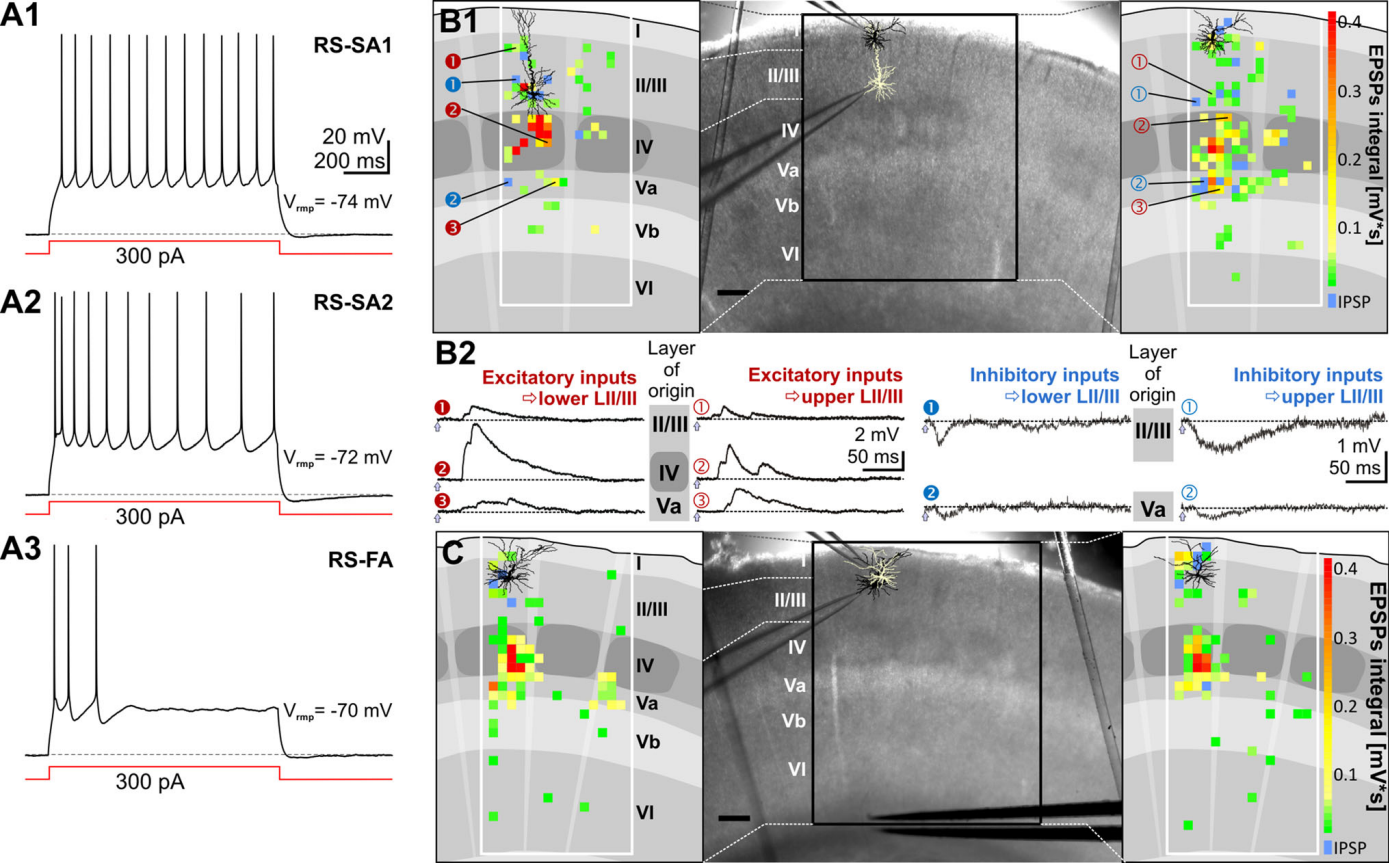
Functional input connectivity differs between pyramidal cells in upper and lower parts of LII/III. **a** Intrinsic electrophysiology: upon depolarizing current injection LII/III pyramidal cells generally showed regular-spiking action potential firing patterns of either slow-adapting type I (RS-SA1, **a1**), slow-adapting type II (RS-SA2, **a2**) or fast-adapting type (RS-FA, **a3**). **b**, **c** Functional input connectivity of pyramidal cells simultaneously recorded in opposite (**b**) or the same part of LII/III (**c**). **b1** Individual functional input maps of a pyramidal cell recorded in the upper third (=upper LII/III, *left panel*) and lower third of LII/III (=lower LII/III, *right panel*). *Middle panel* somatodendritic reconstructions of the two simultaneously recorded neurons superimposed on photomicrographs of the native coronal slice. *Left and right panels* schematic representation of layers, barrels (*dark gray*), columns and septa based on the framed area in the middle panel. Superimposed are the individual reconstructions with a topographic map of their synaptic input origins. EPSPs are color-coded by integral value, IPSPs are blue. Where glutamate stimulation evoked both, only the IPSP is shown. Note the sparse excitatory inputs onto the lower LII/III pyramidal cell originating from LVa. **b2** Representative layer-specific excitatory and inhibitory inputs onto LII/III pyramidal cells. Inputs were recorded in the two pyramidal cells after stimulating locations as depicted in the maps shown in **b1**, at a holding potential (*V*_h_) of −60 mV. **c** Functional input maps of pyramidal cells recorded simultaneously in upper LII/III both show extensive excitatory inputs originating from LIV and LVa. *Scale bar* 200 μm

Slices were transferred to a fixed-stage recording submerged chamber (standard ACSF flow rate of ~1 ml/min at 36 °C) in an upright microscope (Axioskop FS; Carl Zeiss, Germany). The barrel field was visualized at low magnification (2.5×) under bright-field conditions (Fig. 1a) and a target region in the LII/III in vertical register with a LIV barrel was selected for recording. Following visual identification at 40× magnification (40×/0.75 W; Olympus, Germany) using infrared enhanced quarter-field illumination, whole-cell recordings from single or pairs of supragranular pyramidal neurons were performed in current-clamp as well as voltage-clamp controlled current-clamp mode (VCcCC) using two synchronized SEC-05L amplifiers (npi-electronics, Germany). The VCcCC technique ensures stable holding potentials during long time current-clamp recordings by compensation of slow spontaneous changes in the holding potential (integration time > 100 s). Borosilicate glass patch pipettes (electrode resistance 5–7 MΩ) were filled with (in mM): 13 KCl, 117 K-gluconate, 10 K-HEPES, 2 Na_2_ATP, 0.5 NaGTP, 1 CaCl_2_, 2 MgCl_2_, 11 EGTA and 1 % biocytin. Electrophysiological data were not corrected for a junction potential of ca. −10 mV. The signals were filtered at 3 kHz and digitized using an LIH-16000 interface (Heka Elektronik, Germany). Data were recorded, stored and analyzed with PC-based software (TIDA 5 for Windows; Heka Elektronik, Germany). Passive intrinsic electrophysiological properties were tested at resting membrane potential (*V*_rmp_) by applying hyperpolarizing current injection (50 pA), whereas active properties were analyzed by applying depolarizing current injections ranging from 50 to 300 pA at *V*_rmp_. We classified the supragranular pyramidal cells by means of their firing properties into regular-spiking neurons of the slow-adapting type 1 (SA1) and type 2 (SA2), as well as fast-adapting (FA) neurons (c.f. Fig. 2a; Cho et al. 2004; Gottlieb and Keller 1997). This classification was mainly based on the neurons’ ratio of the 3rd and the 9th interspike interval (ISI) (Cho et al. 2004). Neurons of the SA1 type have a 3rd to the 9th ISI ratio of <1.67, those of the SA2 type of >1.67, whereas in FA neurons ceased AP firing before generating the 10th AP. When dual recordings were performed, a second neuron was selected in the same column that was positioned either laterally at a distance of <100 μm or vertically to the first cell at a distance of >200 μm; Figs. 1a1, 2b, c. For the column-wide comparison of neuronal properties, we electrophysiologically classified the excitatory neurons of LIV, LVa and LVb into either regular-spiking or intrinsically burst-spiking neurons, based on criteria described previously (Schubert et al. 2001, 2003, 2006).

### Scanning of glutamate-evoked activity

Our setup and experimental procedures for photolysis of caged glutamate were described in detail previously (Kötter et al. 2005; Schubert et al. 2006). In brief, to map functional connectivity of single neurons, caged glutamate (l-glutamic acid, γ-[α-carboxy-2-nitrobenzyl]ester; Invitrogen, The Netherlands) was added to circulating ACSF (total volume 5 ml), resulting in a 0.5 mM concentration. In control experiments with blocked synaptic transmission, low Ca^2+^/high Mg^2+^ ACSF containing (in mM) 0.2 CaCl_2_ and 4 MgSO_4_ was used. For local stimulation, UV light pulses from a Xenon arc lamp (Rapp OptoElectronic, Germany) were focused on 50 μm × 50 μm large areas. Illumination intensity was calibrated to a value that ensured action potential generation only upon perisomatic photostimulation (i.e., at distances <100 μm) from the soma at *V*_rmp_. In agreement with previous studies (Kötter et al. 2005; Schubert et al. 2001, 2003, 2006), in no control trial recorded at *V*_rmp_, we induced AP firing by stimulation of fields located more than 75 μm vertically above the soma and 50 μm in horizontal register from the soma (*n* = 41 supragranular pyramidal neurons, Fig. 1c). Thus, as described in detail previously (Schubert et al. 2001, 2003, 2006), our calibration allowed (1) mapping at (sub)laminar spatial resolution of at least 75–100 μm and (2) excluded the induction of disynaptic network activation. While mapping the synaptic connectivity, the cell was held at a potential (*V*_hold_) of −60 mV in voltage-clamp controlled current-clamp mode (VCcCC) to reveal hyperpolarizing inhibitory synaptic inputs, in addition to depolarizing excitatory synaptic inputs (Fig. 1b). The intrinsic properties of the recorded cells were controlled before and after termination of each mapping experiment.

### Data acquisition and analysis of glutamate-induced activity

In the recorded neuron, photolysis of caged glutamate induced two main types of activity: (1) Direct responses, induced by activation of glutamate receptors within the recorded cell’s membrane and (2) synaptic inputs induced by activation of presynaptic neurons within the respective flashed field. The properties and reliability of direct responses as well as synaptic inputs upon flash stimulation have been investigated and described in detail before for several other classes of excitatory cortical neurons (Schubert et al. 2006). In agreement with these previous studies, control mappings of pyramidal neurons in LII/III in low Ca^2+^/high Mg^2+^-ACSF (*n* = 8) showed that direct responses occurred only (1) when stimulating fields that contained dendritic extensions of the recorded neuron and (2) at short latency varying between 0.5 ms (perisomatic stimulation) and 6 ms (most distal parts of an apical dendrite; Fig. 1b). These short delays between stimulation and onset of activity reliably distinguished direct responses from flash-induced synaptic inputs; the latter having a delay to onset always longer than 7 ms in LII/III pyramidal cells (Fig. 1b3).

To distinguish between flash-induced activity and spontaneous events, we determined for each cell the total integral value of all spontaneous events within a recording time window of 150 ms. The highest integral value of spontaneous activity obtained in 40 control recordings without stimulation was set as the cell-specific activity threshold. Likewise, following stimulation, for each individual recording, we determined the total integral value of all events within our analysis window (150 ms post-stimulus). If direct responses were induced, their integral values were subtracted from the integral of the synaptic activity (Schubert et al. 2006; see Fig. 1b2). Only excitatory activity that exceeded the cell-specific activity threshold was accepted as a glutamate-induced response, which might result in an underestimation of weak synaptic inputs. All integral values of glutamate-induced synaptic responses were corrected by the mean cell-specific integral value of spontaneous activity. Glutamate-induced responses were analyzed and superimposed on the respective sites of the slice photomicrographs. To quantitatively analyze the connectivity maps, we determined (1) the density and (2) the strength of synaptic inputs arising from a given layer. For determining the density, we calculated the percentage of fields within this layer that delivered excitatory or inhibitory inputs upon flash stimulation separately for the barrel-associated home column (HC), septal columns (SC) and neighboring columns (NC). To determine the strength of induced excitatory inputs, we used the integral value of all EPSPs within the post-stimulus time window of 150 ms.

### Histological procedures

After recording, slices were fixed in phosphate-buffered 4 % paraformaldehyde for 24 h at 4 °C. For visualization of the biocytin-filled neurons, slices were processed as described previously (Staiger et al. 2004). Furthermore, for most of the slices, we used an additional silver/gold intensification following the protocol of Bender et al. (2003). The barrel field was either visualized by cytochrome oxidase histochemistry, or the barrel pattern of the micrograph of the native slice was transferred manually into the reconstruction. Reconstruction and morphological analysis of the biocytin-labeled neurons were made using a Nikon Eclipse 800 (Nikon, Germany) attached to a computer system (Neurolucida; MBF Bioscience Europe). Data were not corrected for tissue shrinkage. However, from several measurements we have estimated the shrinkage to be around 10–15 % in *x*-/*y*-axes and 40–60 % in the *z* axis. The reconstructed cells were (1) superimposed onto the photomicrograph of the native slice using standard graphics software and (2) quantitatively analyzed with Neuroexplorer (MBF Bioscience Europe).

### Statistical analysis

For assumption-free comparison of neuronal properties across a cortical column, in a first step, we performed a classical sliding window analysis of excitatory neuronal of cortical layers II/III to LVb. For each individual neuron, we determined the relative vertical position within a column by quantifying the distance between the LVa-IV border and the pia. The LIV-Va border was assigned to the 0 % position, the pia to 100 % and positions within the infragranular layers to negative values accordingly (see Fig. 5a). We performed the sliding window analysis of individual functional (input connectivity) and structural (somatodendritic) properties at a window span and step size of 10 % of the relative distance between LIV-Va border and the pia. At this step size, each window contained data of a minimum of 5 neurons.

In a second step, we tested the general structural and functional similarity of neurons by performing an unsupervised hierarchical cluster analysis using Ward’s linkage method. We only included parameters of which data were available for neurons of all layers i.e., subsets of somatodendritic and functional input connectivity properties. The functional properties included in this analysis were: layer-specific density of excitatory synaptic inputs originating from LII/III, LIV, LVa, LVb and LVI of the home column and the neighboring column, layer-specific density of inhibitory synaptic inputs originating from home column LII/III, LIV, LVa (no consistent quantitative data were available for inputs from LVb and LVI) and total density of excitatory as well as inhibitory inputs from the home column. As structural data, we furthermore considered the following somatodendritic properties: (1) total number of endings, (2) length of the apical dendrite, (3) total number of dendrites and (4) maximal trunk diameter of the apical dendrite. Sufficient quantitative axonal data were not available for the entire set of neuronal populations.

To analyze to which extent neurons in LII/III can be considered as populations with statistically similar input/output properties, in a third step we performed an adapted sliding window analysis in which we compared the properties of one neuron population with a population that was becoming increasingly distant from the first one. For this analysis, we assigned the relative vertical position of the recorded somata within LII/III, (LI border = 0 %; LIV border = 100 %) and tested from which vertical position in LII/III neuron populations differed structurally and functionally significantly from a reference population at the upper and lower limits of LII/III, i.e., a population at the LI or LIV border. This multiparametric analysis (MANOVA, Bonferroni corrected) included sets of dendritic, axonal, intrinsic electrophysiological and synaptic input properties that showed significant correlation with the relative soma position within LII/III. The adapted sliding window analysis was performed with windows of 20 % span of the relative distance between LIV-II/III border and pia. This span within LII/III covered a similar proportion of the cortical column as the window span used for the classical sliding windows analysis of the entire cortical column. For each set of parameters, the sliding windows were moved in steps of 5 % from a base window at the LI border downwards to LIV; and, vice versa, from a base window at the LIV border upwards to LI. We defined the lower boundary of where the reference population in upper LII/III becomes statistically dissimilar from their counterparts below, i.e., where the MANOVA comparison dropped below *p* = 0.05 in the downward sliding window approach. Likewise, in the upward approach, we defined the upper boundary of lower LII/III by a drop below *p* = 0.05. To obtain a concrete number for the depth of these statistical borders, sigmoidal functions (Yin et al. 2003) were fit to each of these two sets of *p* values: $P(d) = \left( {1 + \frac{{d_{e} - d}}{{d_{e} - d_{m} }}} \right)\left( {\frac{{d - d_{b} }}{{d_{e} - d_{b} }}} \right)^{{\left( {\frac{{d_{e} - d_{b} }}{{d_{e} - d_{m} }}} \right)}}$, with *d* indicating depth. To obtain a decreasing sigmoidal function, we set *d* → 100 % − *d* in this formula. The parameters indicate the depths for the beginning *d*_b_, maximum change *d*_m_, and end *d*_e_ of the sigmoidal growth. We also calculated bands by scanning the entire sigmoid parameter space, i.e., *d*_b_ ≤ *d*_m_ < *d*_e_, where *d*_b_ can vary from 0 to just less than 100 %. We took steps of 0.1 % in each parameter, resulting in a total of 167 million sigmoidal functions trialed. The bands calculated this way provide a visual representation of the uncertainties of the fit.

For analysis of the properties of the three neuronal populations that were defined by the adapted window analysis (i.e., equally sized parts of LII/III: upper, middle and lower LII/III), we performed a multiparametric discriminant analysis. The analysis was done independently for structural and functional properties. Statistical analysis of individual parameters was performed using a linear regression (Pearson correlation) and a multivariate analysis of variance (MANOVA) with layer-specific data as repeated measures and Bonferroni correction for post hoc pairwise comparisons (SPSS 9; SPSS Inc.). If not mentioned differently, data are presented as mean ± SEM.

### Construction of average functional maps

We also applied a novel procedure to illustrate the average functional connectivity of neuronal populations in upper, middle and lower LII/III, including a visualization of the confidence levels of evoked synaptic inputs. For this method, the individual inputs maps were projected into a template map by matching the slice-specific laminar and columnar cytoarchitecture with the template using vertical and horizontal scaling. This procedure involved, as a first step, translating, scaling and rotating photomicrographs of the native slice in a graphics program until an optimal match with a template’s center barrel position, overall barrel size, slice orientation and pial surface was obtained. For every template grid point, we then calculated the corresponding position in the original photomicrographs, and hence in the glutamate-induced activity maps, by mathematically inverting the used transformations. Since the reorientation and scaling procedure used only linear transformations and translation, this inversion was exact. Subsequently, we averaged the activity found in the corresponding glutamate-induced activity maps and assigned these averages $\bar{x}$ to the template grid points, displaying only average values for which significant experimental evidence had been obtained. To ensure that our maps were not marred by the varying number of contributing slices for different template grid points, we estimated individual confidence levels that the averaged activity $\bar{x}$ at a given point differed from zero with $\zeta = 2\text{T} \left( {\frac{{\bar{x}\sqrt n }}{\sigma },n} \right) - 1$, where *n* was the number of contributing slices, *σ* the sample standard deviation, and T the cumulative Student’s *t* distribution. For display purposes, only those template grid points to which at least three slices contributed (*n* ≥ 3) and for which $\zeta \ge 68.3\,\%$ (zero activity excluded by “one sigma”) were considered further.

## Results

Previous studies (Bureau et al. 2006; Shepherd and Svoboda 2005) imply that pyramidal neurons in the supragranular layers II/III (LII/III) of the rodent barrel cortex show different morphological and function properties, depending on whether they are located in the upper part of LII/III, i.e., close to LI, or in the deeper part, i.e., close to LIV. Here, we investigated whether and how the input–output properties of pyramidal cells are related to their somatic position in LII/III and whether there are indications for definable laminar borders in LII/III. We used slice preparations of juvenile rats containing the barrel cortex, and focused on supragranular pyramidal neurons in a barrel-related column. All 162 neurons included in our study were filled with biocytin and morphologically classified as pyramidal cells having a resting membrane potential (*V*_rmp_) more negative than −60 mV. In a first step, to describe general differences between neurons that are located closer to LI versus those closer to LIV, we horizontally segregated LII/III into three equally sized parts, i.e., upper LII/III, middle LII/III and lower LII/III. Given that in our acute slice preparations the distance between the border to LI and LIV was approximately 400 μm (Fig. 1), each part had a vertical extent of 130–140 μm.

### Electrophysiology and general functional input connectivity of LII/III pyramidal neurons

Supragranular pyramidal neurons (*n* = 162) belonged to one of the three different subclasses of regular-spiking neurons: slow-adapting types 1 (SA1) and 2 (SA2), and fast-adapting (FA) (Cho et al. 2004, Fig. 2a, for details see “Materials and methods”). Whereas SA1 and SA2 firing patterns were observed throughout LII/III, FA neurons were absent in the upper LII/III (Table 1).

**Table 1:** Electrophysiological properties of supragranular pyramidal neurons

Properties	Upper II/III	Middle II/III	Lower II/III	Depth corr.
	(*n* = 60)	(*n* = 47)	(*n* = 55)	*R* (*n* = 162)
Passive intrinsic				
*V*_rmp_ (mV)	−69.3 ± 0.9	−69.4 ± 0.7	−70.9 ± 0.8	−0.15
*R*_m_ (MΩ)	110.2 ± 5.8***	86.1 ± 4.9**^1^	77.7 ± 3.0	−0.49**
*τ*_m_ (ms)	18.7 ± 0.7*	18.1 ± 1.3	14.9 ± 0.6	−0.3**
Active intrinsic				
AP threshold^1^ (mV)	−47.7 ± 0.6*	−47.7 ± 0.7*^2^	−49.9 ± 0.5	−0.12
AP amplitude (mV)	78.5 ± 1.1	79.5 ± 1.3	82.6 ± 1.2	0.13
AP halfwidth (ms)	1.7 ± 0.1**	1.5 ± 0.1*^1^	1.5 ± 0.1	−0.27**
1st ISI-weak^1^ (ms)	151.9 ± 9.7**	108.7 ± 5.9**^1^	102.0 ± 7.6	−0.32**
1st ISI-strong^2^ (ms)	54.8 ± 3.0**	47.2 ± 3.2**^1^	44.7 ± 3.2	−0.22*
Firing pattern^2^				
RS-SA1 type	82.4 %	71.7 %	61.4 %	
RS-SA2 type	17.6 %	23.9 %	27.3 %	
RS-FA type	0 %	4.3 %	11.4 %	

Data are mean ± SEM. Active properties were measured: ^1^ just suprathreshold, eliciting 2–4 APs, or ^2^ by stronger depolarizing currents, eliciting 10–14 APs. Asterisks mark significant differences upper LII/III vs. lower LII/III (*), upper LII/III vs. middle LII/III (*^1^) and lower LII/III vs. middle LII/III (*^2^): MANOVA, Bonferroni corrected, * p < 0.05, ** p < 0.01, *** p < 0.01. “Depth corr.” is the Pearson correlation of a parameter with relative soma position (0 % = border LI-LII/III, 100 % = border LII/III-LIV). Firing patterns were classified by the adaptation ratio (9th/3rd ISI): ≤1.67 RS-SA1 vs. >1.67 RS-SA2. Neurons that stopped firing were classified as fast-adapting RS-FA

We investigated the layer-specific distribution of intracortical excitatory and inhibitory inputs of supragranular pyramidal neurons (*n* = 44) at *V*_h_ = −60 mV using multisite focal photolysis of caged glutamate in combination with whole-cell recordings of single (*n* = 24) or simultaneous recordings of two neurons (*n* = 10). In the latter case, we recorded and mapped two pyramidal neurons at similar cortical depth within LII/III (*n* = 5) or at different cortical depth (vertical distance > 200 μm; *n* = 5; see Fig. 2b, c). These simultaneous recordings allowed us to directly observe differences between neurons of different supragranular positions under identical experimental conditions. In agreement with previous studies, after identifying and excluding spontaneous as well as direct responses (see “Materials and methods”), this procedure produced detailed maps with (sub)laminar spatial resolution (~75 μm) showing monosynaptic origins for excitatory and inhibitory inputs onto the recorded neurons (Figs. 2b, c, cf. 3, Schubert et al. 2001, 2003, 2006).

**Fig. 3:**
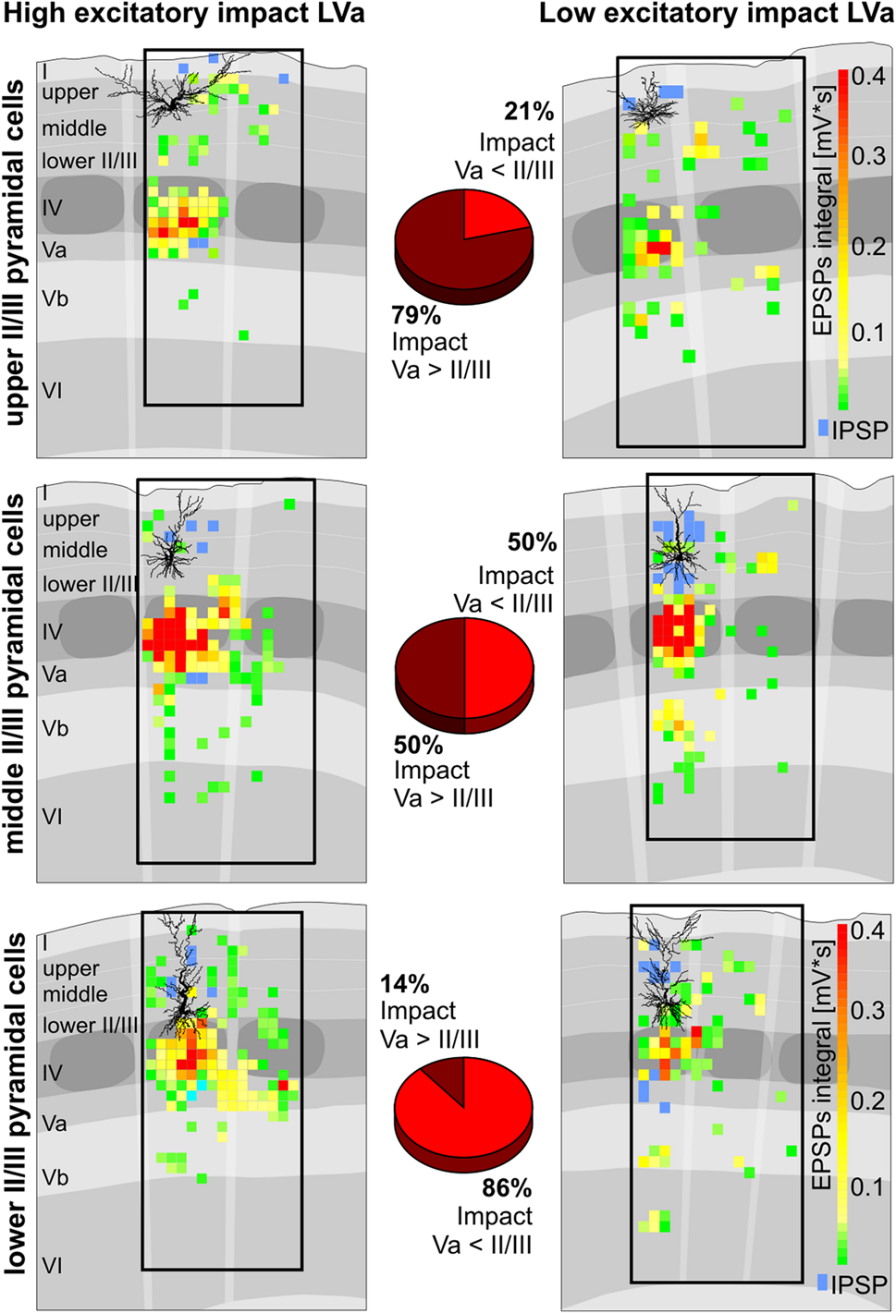
Pyramidal neurons with sparse or prominent LVa inputs show depth-dependent distribution within LII/III. Impact (density of inputs × average strength) of excitatory inputs from LII/III vs. LVa. Throughout LII/III (*upper row* upper LII/III, *middle row* middle LII/III, *lower row* lower LII/III) a subset of neurons received intracolumnar excitatory inputs from LVa which had either a higher (*left side*) or lower impact (*right side*) than local inputs from LII/III. Pie charts show the proportion of these two subsets in dependency of the position within LII/III (upper LII/III *n* = 14; middle LII/III *n* = 16; lower LII/III *n* = 14)

In general, supragranular pyramidal neurons received most of their synaptic inputs from within their home column. Stimulus-evoked excitatory postsynaptic potentials (EPSPs) originated most prominently from the supragranular compartment itself, but also from LIV (Fig. 2b1, c). Excitatory inputs from LIV of the home column often consisted of numerous EPSPs of high amplitudes (>2 mV) and, as a summation of excitatory inputs, of high integral values (>0.1 mV/s) within a time window of 150 ms post-stimulus.

A striking difference between neurons in lower LII/III as compared to those in upper LII/III, was that the latter, in 11 out of 14 cases, received prominent excitatory input from LVa, the main cortical target layer of the paralemniscal pathway. We weighted the functional impact of the excitatory inputs originating from a given layer by multiplying the layer-specific density and the average strength of excitatory inputs. We found that lower LII/III possessed mainly neurons in which the impact of local inputs exceeded that of LVa (12 out of 14 neurons; Figs. 2b, 3). Gradually, towards middle and upper LII/III, the occurrence of these neurons became sparse. Whereas in middle LII/III still half of the neurons (8 out of 16) showed higher impact local inputs, the upper LII/III possessed mainly neurons in which the impact of LVa inputs exceeded that of local ones (11 out of 14; Figs. 2b, 3). Hyperpolarizing inhibitory synaptic potentials (IPSPs) were induced less frequently, namely in about 10 % of the fields that delivered synaptic inputs. IPSPs originated mainly from the intracolumnar domains of LI to lower II/III, but also from LVa (Fig. 2b2). The strength of induced excitatory inputs per layer was typically heterogeneous and could range from weak (integral values <0.05 mV s) to strong (≥0.1 mV s) inputs. For reasons described previously (Schubert et al. 2001), we did not determine the strength of inhibitory inputs. The inhibitory functional input connectivity appeared similar for neurons throughout LII/III.

### General somatodendritic structure and output connectivity of LII/III pyramidal cells

**Fig. 4:**
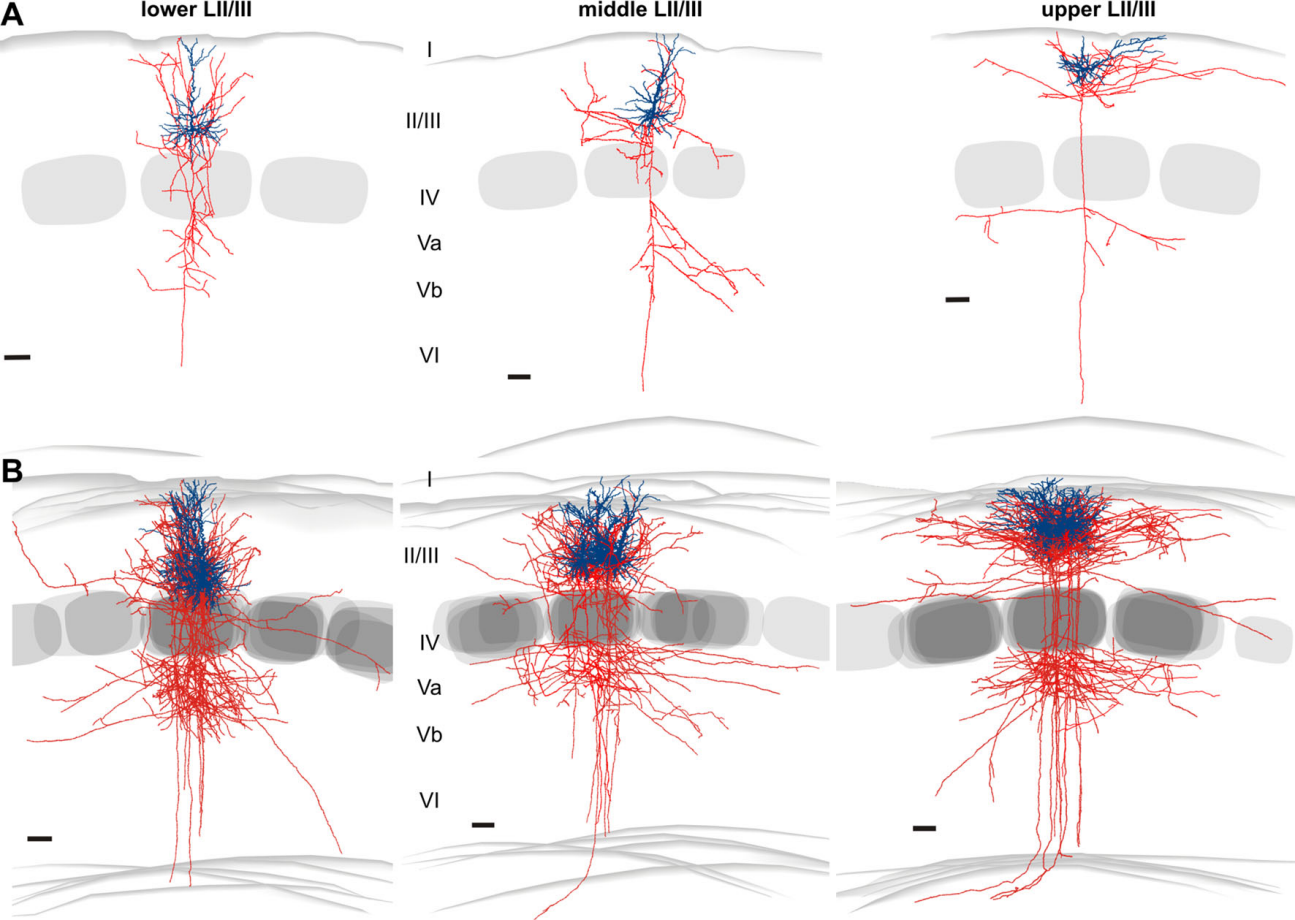
Morphological properties of LII/III pyramidal neurons show depth-dependent changes. **a** Reconstructions of pyramidal neurons in upper, middle and lower LII/III; somata and dendrites in *dark blue*, axons in *red*. *Gray areas* in LIV illustrate barrels. *Lower panels* six neurons superimposed by aligning their home barrels; *upper panels* one example neuron. *Scale bars* 100 μm

Our morphological data are based on reconstructions of the somatodendritic domain of 59 neurons and of the intracortical axonal projections of 22 well-preserved neurons. All pyramidal cells possessed several (2–7) basal dendrites that emerged from an ovoid to pyramidal shaped soma and an apical dendrite that always reached LI (Fig. 4). Two main groups of pyramidal cells were recognizable: those with “atypical” (oblique) apical dendrites and those with “typical” (straight) ones (Fig. 4a). Typical pyramidal cells could be found throughout LII/III, whereas the atypical ones were detected nearly exclusively (18 out of 19) in upper LII/III. In terms of the output connectivity, a common feature of all neurons was that the main axonal stem descended toward or into the white matter, giving rise to a local plexus as well as several more distant horizontal or recurrent collaterals. The main terminal fields of these intracortical collaterals, apart from the ones found within the supragranular layers, were the infragranular LVa and LVb. Interestingly, for lower LII/III pyramidal cells, also LIV of the home column was a prominent target (HC, Fig. 4). Unlike pyramidal cells in lower LII/III, those in upper LII/III typically traversed LIV with few (if any) collateral branches. Besides this difference, pyramidal neurons in lower LII/III showed axonal projections that were as a whole more restricted to their respective home column than those in the upper LII/III, although some horizontal or oblique collaterals projected to the neighboring septa or barrel columns. Neurons in middle LII/III showed axonal projection patterns that appeared to be in between that of the upper and lower LII/III (Fig. 4).

### Structural and functional input/output connectivity of excitatory neurons can define layer borders

**Fig. 5:**
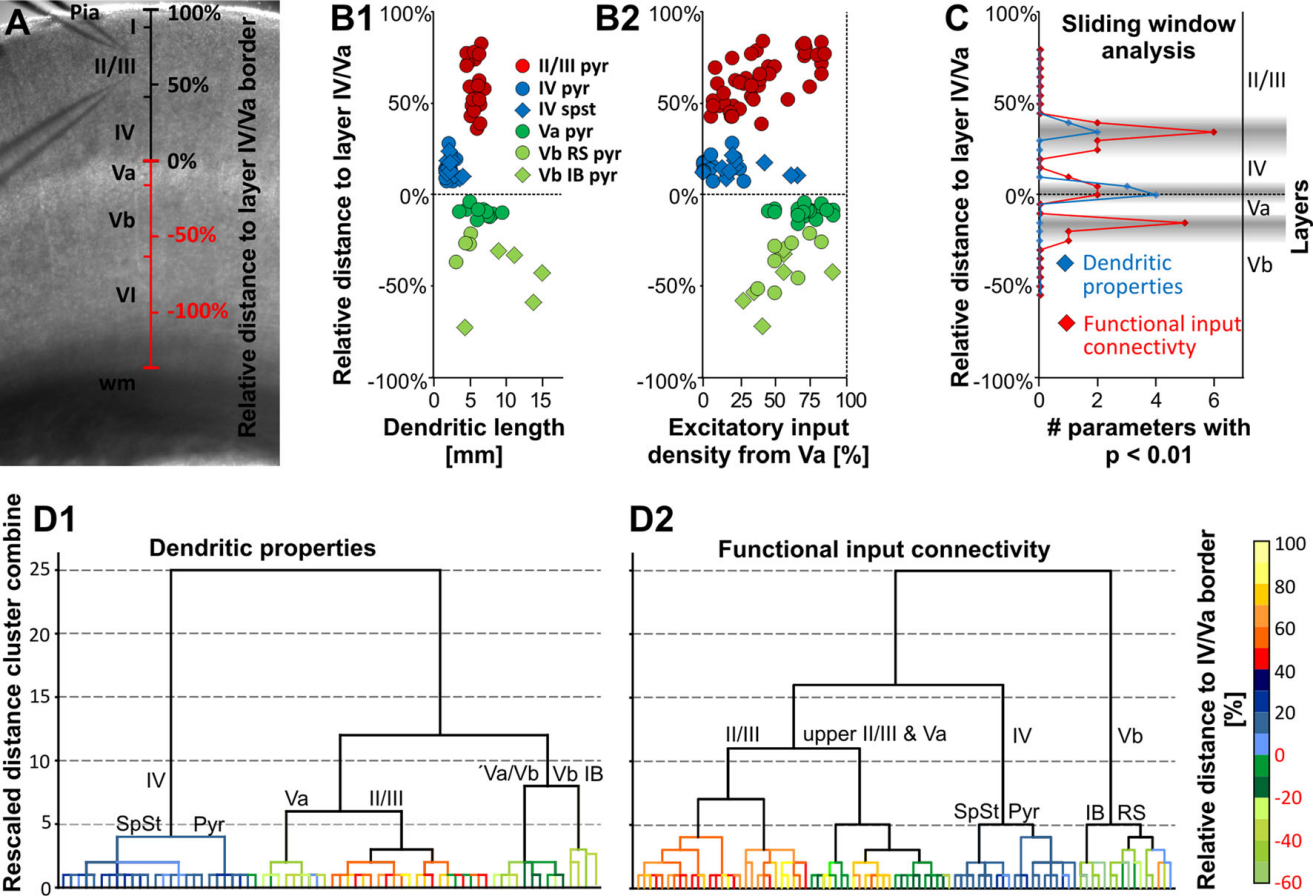
Changes in functional and structural properties of excitatory neurons mark borders between established cortical layers but not within LII/III. **a** Native slice image illustrating the designation of the relative vertical soma position of recorded excitatory neurons in S1 cortex. The distance between pia and the LIV-Va border was set to 100 % with the latter being set to 0 %. Accordingly, positions in the infragranular layers were assigned to negative percentages. **b** Examples of structural (**b1**) and functional properties (**b2**) in relation to the relative soma position of excitatory neurons recorded in LII/III to LVb (LII/III *n* = 44; LIV *n* = 24; LVa *n* = 27; LVb *n* = 15). Data points are coded for home layer and subclass of the recorded excitatory neurons. **c** Classical sliding window analysis (window span and step size 10 %) showing at which relative soma position individual structural and functional parameters (tested functional properties *n* = 15, structural dendritic properties *n* = 5) change significantly between adjacent windows. *Gray shaded areas* mark the range in which individual brain slice cytoarchitecture revealed layer borders. **d** Dendrograms following unsupervised hierarchical cluster analysis of excitatory neurons of cortical LII/III to LVb based on 15 functional properties and 5 structural properties (Ward’s linkage). The color code marks the relative position of the recorded neurons, labels in the diagrams highlight the dominant cell type found in a cluster. *SpSt* spiny stellate neuron, *RS* *pyr* regular-spiking pyramidal cell, *IB pyr* intrinsically bursting pyramidal cell

Our data, so far described, confirm and greatly extend the assumption that LII/III pyramidal cells show significant differences in their input/output connectivity, dependent on whether they are located in the upper LII/III or lower LII/III. We tested whether the underlying structural and functional properties change in a way that would support the presence of one or more distinct layers within LII/III. Previous studies suggest that, within a layer, neurons of a particular morphological class share input/output properties (Feldmeyer 2012; Schubert et al. 2007; Thomson and Lamy 2007). We performed a sliding window analysis that compared structural and functional properties of adjacent neuron populations within a certain span of the cortical depth. To increase the validity of this test, we extended this analysis by including excitatory neurons of LIV (*n* = 24), LVa (*n* = 27) and LVb (*n* = 15) in addition to the pyramidal neurons of LII/III (*n* = 44). The relative position of all somata was determined in relation to the LIV-Va border (for details see “Materials and methods”, Fig. 5a). For this analysis (into which LVI is not included because it has not been studied by us so far), we focused on the somatodendritic structure and functional input connectivity since these parameters were available for the entire data set in a quantified manner. A list of the parameters used in this analysis is provided in “Materials and methods”. Depending on the relative soma position, most functional and structural properties of the neurons of a certain layer showed abrupt changes at one or more established laminar borders (relative border positions LII/III-LIV 35–40 %, LIV-LVa 0 %, LVa-Vb −15 to 20 %; Fig. 5b). In LII/III, such changes were absent. In agreement with this, the classical sliding window analysis at a window width of 10 % showed that at every established layer border, a number of functional properties changed significantly (*p* < 0.01, Fig. 5c), most consistently, in terms of layer-specific intracolumnar and transcolumnar density of excitatory inputs. However, there was no single functional property that showed significant changes at every classical border. The analysis of the structural properties failed to detect the LVa-Vb border although all tested dendritic properties changed at the LII/III-IV and LIV-Va border, respectively. Within LII/III, significant changes were absent. Furthermore, unsupervised hierarchical cluster analysis of the dendritic properties showed that the population of LII/III neurons was not found in separate clusters (Fig. 5d1). Analysis of the 15 functional properties revealed a subset of upper LII/III neurons that formed a cluster with LVa pyramidal neurons. Relevant properties for this clustering were the excitatory inputs from LIV and LVa, which for both of these neurons reflect a main source of input (c.f. Schubert et al. 2006).

### Statistically similar populations of pyramidal neurons in LII/III

**Fig. 6:**
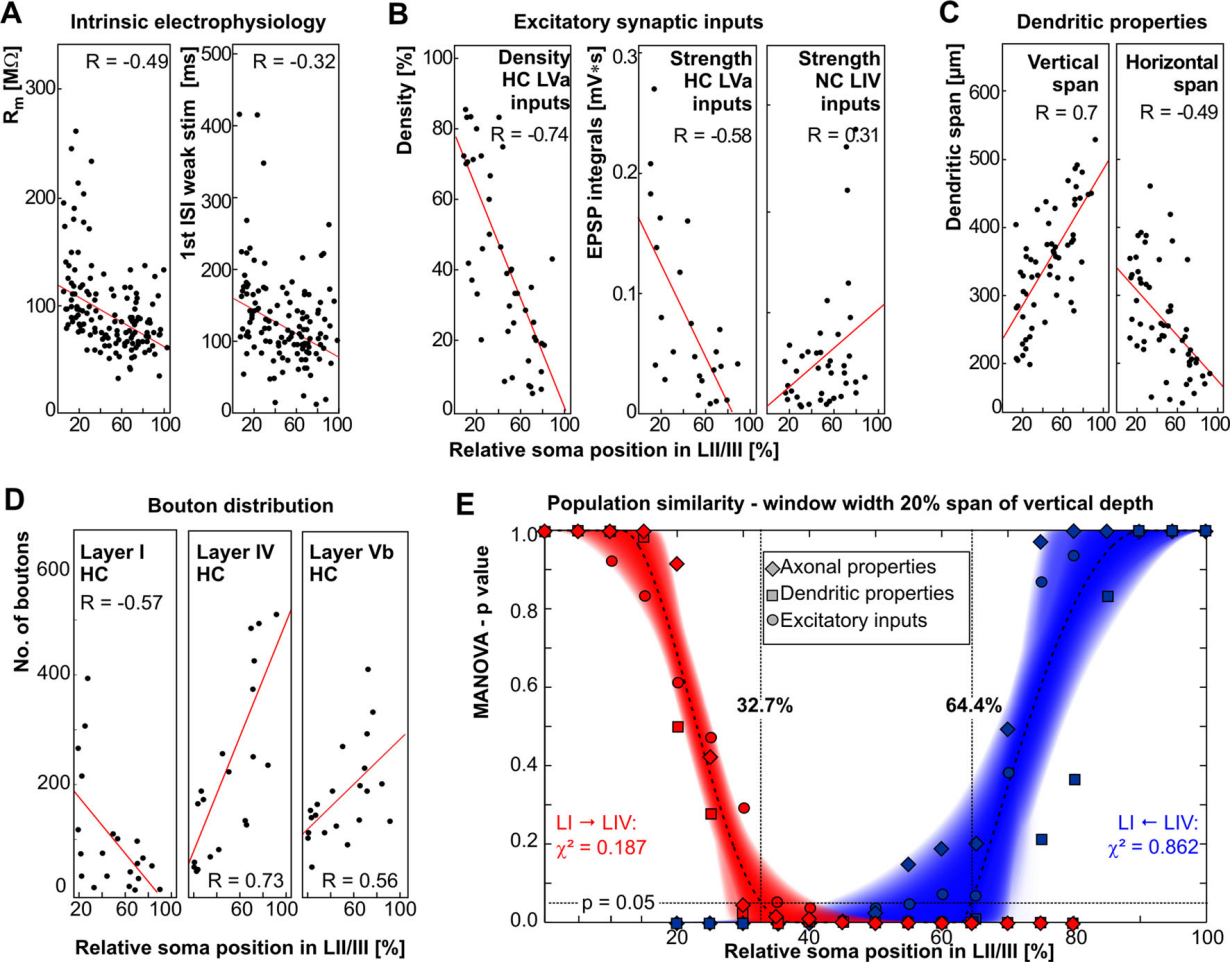
Properties of individual pyramidal cells correlate with soma position in the supragranular LII/III. Depth has been scaled to range from 0 % (border to LI) to 100 % (border to IV). *Red lines* illustrate linear correlations. **a** Intrinsic electrophysiology: membrane-resistance (*R*_m_) and 1st interspike interval (ISI) to just suprathreshold stimulation (*n* = 162; *R*_m_: *p* < 0.001, 1st ISI: *p* < 0.001). **b** Functional input connectivity: excitatory synaptic input density and strength (upper II/III *n* = 14, middle II/III *n* = 16, lower II/III *n* = 14; input density HC LVa *p* < 0.001, input strength HC LVa *p* < 0.001, NC LIV *p* < 0.05). **c** Structural input properties (dendritic properties, *n* = 59; Pearson correlation *p* < 0.001 each) and **d** output properties (axonal properties, *n* = 22; home column LI and LIV *p* < 0.001, LVb *p* < 0.01). **e** On the neuronal population level, a modified sliding window analysis illustrates statistical similarity of neuronal properties within upper and within lower LII/III. Neuronal population properties of a base window at the *top* (0–20 %, *red*) and *bottom* (80–100 %, *blue*), respectively, are compared with an equally sized sliding window. Sigmoidal fits to the MANOVA *p* values are shown as *black lines* (best fit) and bands (uncertainty for 50 % increase of *χ* ^2^). Boundary values for upper LII/III at 32.7 % and lower LII/III at 64.4 %, respectively, derive from the intersection of the best fit sigmoids with *p* = 0.05 (*vertical/horizontal dashed lines*). *HC* home column, *NC* neighboring column

The classical sliding window analysis of our data does not support the notion that LII/III contains laminar borders. However, we found that several structural and functional properties changed gradually within LII/III resulting in significant linear correlation (Pearson correlation) with respect to soma position. These properties were found among intrinsic electrophysiological properties, functional input connectivity as well as structural input and output connectivity (examples are illustrated in Fig. 6a–d). A summary of the tested intrinsic electrophysiological data is provided in Table 1, for morphological data see Tables 2 and 3. In terms of functional input connectivity, we found a significant linear correlation for density and strength of excitatory inputs originating from LVa of the home column (density *R* = −0.74, *p* < 0.001; strength *R* = −0.58, *p* < 0.001) as well as for the strength of excitatory inputs originating from LIV of the neighboring column (*R* = 0.31, *p* = 0.038). The linear change of neuronal properties with respect to depth implies that these individual properties are indeed heterogeneous in LII/III. Therefore, depending on where in LII/III pyramidal neurons and their respective networks are positioned, they may serve different functions in intracortical sensory signal processing. To quantitatively assess the input/output connectivity of LII/III neuronal networks, we tested up to which position within LII/III, neuron populations of the upper and the lower LII/III can still be considered as statistically similar. For this purpose, we performed an adapted sliding window analysis on the different sets of somatodendritic, axonal, intrinsic electrophysiological and functional input parameters (tested properties are given in Material and Methods). We tested for differences both in the LI → LIV and LIV → LI direction (see “Materials and methods” for details). In the parameter sets, we included only those parameters that correlated significantly with vertical depth. The resulting *p* values (MANOVA, Bonferroni corrected) reflect the similarity between the neural population in the base windows and the one in the sliding window. Base window and starting point for the sliding window were at the border to LI (0 % vertical depth) or at the border to LIV (100 % vertical depth), respectively. We did not find extended plateaus of similar *p* values in any set of tested parameters, except where the test window overlapped extensively with the base window at the upper and lower supragranular borders (Fig. 6e). Using *χ* ^2^ fits of sigmoids on the calculated MANOVA *p* values for the individual sets of parameters, we could determine the vertical depth at which the neuron populations within the sliding window became significantly different from the respective base window populations.

**Table 2:** Morphological properties of supragranular pyramidal neurons

Properties	Upper II/III	Middle II/III	Lower II/III	Depth corr.
Somatodendritic	(*n* = 20)	(*n* = 19)	(*n* = 20)	(*n* = 59)
Horizontal soma diameter (μm)	15.4 ± 0.3	16.1 ± 0.8	15.6 ± 0.5	−0.1
Vertical soma diameter (μm)	18.3 ± 0.5**	20.6 ± 0.7*^1^	22.0 ± 0.6	0.36**
Soma area (μm^2^)	177.7 ± 6.7	214.0 ± 18.0	214.6 ± 11.8	0.1
Vertical dendritic span (μm)	281.2 ± 12.6**	372.7 ± 14.6*^1^	394.9 ± 17.1	0.7***
Horizontal dendritic span (μm)				
Apical	319.0 ± 15.8**	262.1 ± 15.8*^1^	214.7 ± 13.0*^2^	−0.49**
Basal	215.8 ± 7.1	218.1 ± 7.5	214.7 ± 13.0	0.06
No. of primary dendrites (*n*)	5.9 ± 0.3	4.9 ± 0.2	5.2 ± 0.2	−0.3*
Max. dendritic diameter (μm)				
Basal	2.4 ± 0.1	2.6 ± 0.1	2.9 ± 0.2	0.32**
Apical	3.9 ± 0.2	3.9 ± 0.2	4.9 ± 0.2	0.03
Length basal dendrites (μm)	2,664 ± 162	2,680 ± 148	2,919 ± 136	0.11
Length apical dendrites (μm)	2,874 ± 153	2,856 ± 145	2,791 ± 134	0.02
No. of dendritic endings (*n*)				
Basal	35.4 ± 1.5	29.7 ± 1.7	32.9 ± 1.6	−0.25
Apical	27.9 ± 1.8	26.4 ± 1.3	25.6 ± 1.1	−0.13

Axonal	*n* = 9	*n* = 6	*n* = 7	*R* (*n* = 22)
Total axonal length (μm)	10,695 ± 558	11,268 ± 1,239	11,332 ± 812	0.15
Total bouton No. (*n*)	1,999 ± 167	1,930 ± 263	1,887 ± 220	−0.13
Bouton density (*n*/100 μm)	18.5 ± 0.8	17.1 ± 1.3	16.5 ± 0.9	−0.2

Data are mean ± SD. Asterisks mark significant differences in upper LII/III vs. lower LII/III (*) and in upper LII/III vs. middle LII/III (*^1^): MANOVA, Bonferroni corrected, * *p* < 0.05, ** *p* < 0.01, *** *p* < 0.01. “Depth corr.” is the Pearson correlation of a parameter with relative soma position (0 % = border LI, 100 % = border LIV)

**Table 3:** Layer- and column-specific bouton distribution

Nr. of boutons	Upper II/III	Middle II/III	Lower II/III	Depth corr.
	(*n* = 9)	(*n* = 6)	(*n* = 7)	*R* (*n* = 22)SW
Home columns				
LI	183 ± 40**	49 ± 16*^1^	41 ± 13	−0.57**
LII/III	705 ± 82	675 ± 83	573 ± 93	−0.21
LIV	95 ± 20***	225 ± 67*^2^	392 ± 43	0.73***
LVa	254 ± 35	260 ± 46	317 ± 99	0.09
LVb	136 ± 15*	153 ± 25*^2^	252 ± 36	0.56**
LVI	79 ± 16	56 ± 12	37 ± 9	−0.33
Neighboring columns				
LI	88 ± 28*	14 ± 10	5 ± 3	−0.44
LII/III	188 ± 52*	99 ± 24	50 ± 25	−0.38
LIV	21 ± 17	42 ± 33	29 ± 12	0.28
LVa	50 ± 22	134 ± 54	40 ± 18	−0.25
LVb	33 ± 14	65 ± 29	51 ± 21	0.14
LVI	10 ± 7	0 ± 0	7 ± 7	0.01
Septal columns				
LI	13 ± 3*	8 ± 3	2 ± 1	−0.44*
LII/III	46 ± 9	66 ± 19	20 ± 6	−0.16
LIV	8 ± 5	14 ± 7	10 ± 4	0.15
LVa	15 ± 4	33 ± 7**^2^	6 ± 3	−0.13
LVb	11 ± 3	10 ± 4	15 ± 6	0.09
LVI	0 ± 0	0 ± 0	1 ± 1	0.18

Data are mean ± SEM. Asterisks mark significant differences between the neuron populations in upper LII/III vs. lower LII/III (*), upper LII/III vs. middle LII/III (*^1^) and lower LII/III vs. middle LII/III (*^2^): MANOVA, Bonferroni corrected, * *p* < 0.05, ** p < 0.01, *** p < 0.001. “Depth corr.” is the Pearson correlation of a parameter with relative soma position (0 % = border LI-LII/III, 100 % = border LII/III-LIV)

In the LI → LIV direction, neuronal populations became significantly different (i.e., *p* < 0.05) from the base population at depths between 27.0 and 35.4 % depending on analysis window size and respective set of parameters (best fit, Fig. 6e). In the LIV → LI direction, differences reached significance at depths between 57.0 and 74.9 %, except for the intrinsic electrophysiological properties. Combined *χ* ^2^ fits on all neural properties (excluding the intrinsic electrophysiological ones), yielded 32.7 % (LI → LIV) and 64.4 % (LIV → LI), respectively. Taking into account the variation across individual sets of parameters and fit uncertainties (shown as bands in Fig. 6e), our data allow a separation of LII/III into three equally sized parts. Therefore, for further quantitative analysis of the input/output connectivity of pyramidal cell populations in LII/III, we used the segregation into lower, middle and upper LII/III as we previously had introduced them for the general comparison between neurons in different locations within LII/III. Note that because of the gradual change of input/output properties, the neurons within each part still have to be considered as heterogeneous.

### Functional input properties of neuronal populations in lower, middle and upper LII/III

**Fig. 7:**
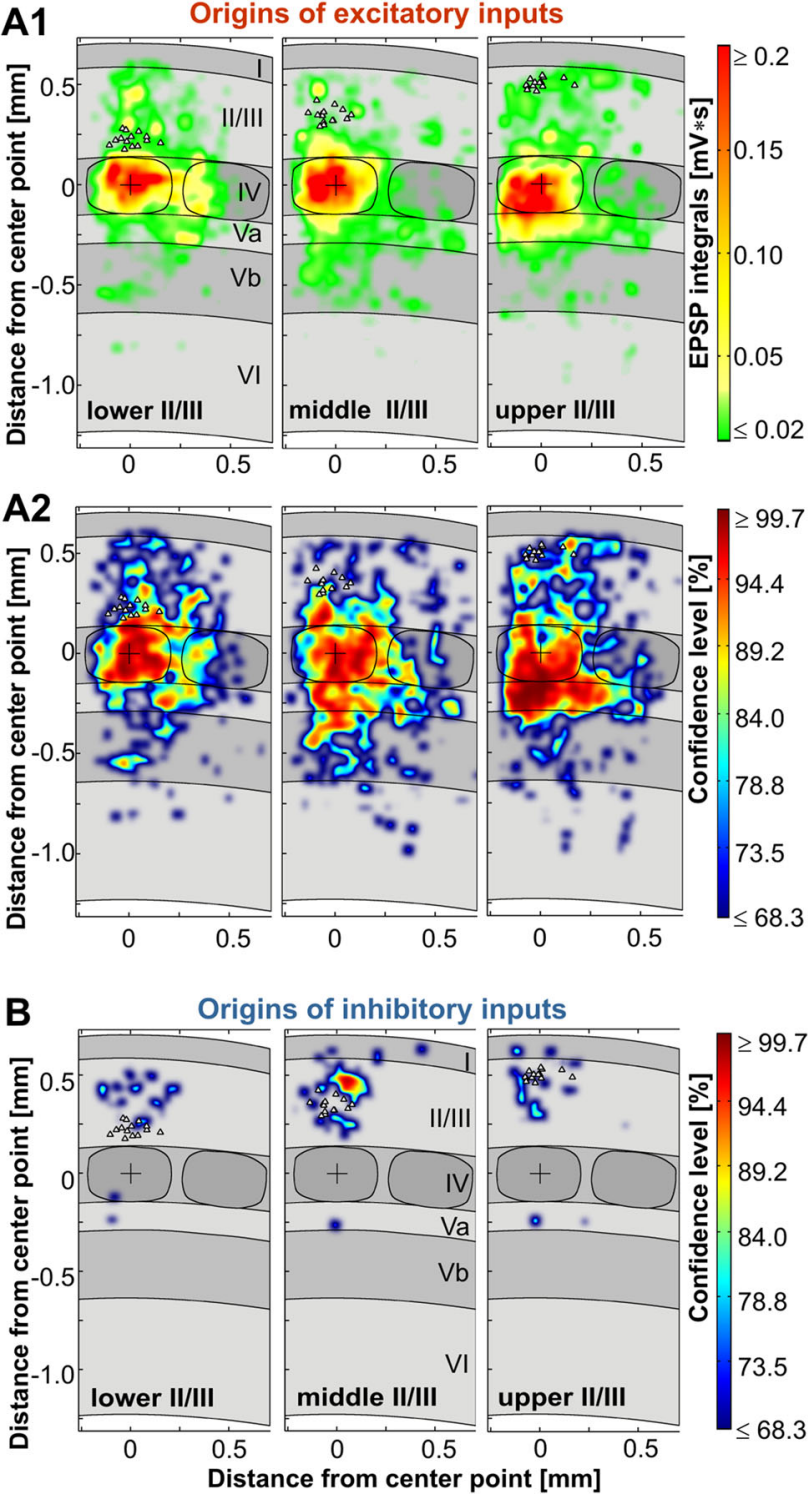
Excitatory and inhibitory input connectivity patterns of neural populations in upper, middle and lower LII/III. Averaged connectivity maps are constructed by linearly transforming individual maps (upper LII/III *n* = 14; middle LII/III *n* = 16; lower LII/III *n* = 14) to the cortical template shown as *gray* background. *White triangles* label the positions of the recorded neurons, a cross the alignment center in the home barrel. **a1** Averaged strength of excitatory inputs with confidence levels ≥68.3 %. **a2** Confidence levels for the origins of excitatory inputs. **b** Averaged maps constructed for the confidence levels of inhibitory inputs

**Fig. 8:**
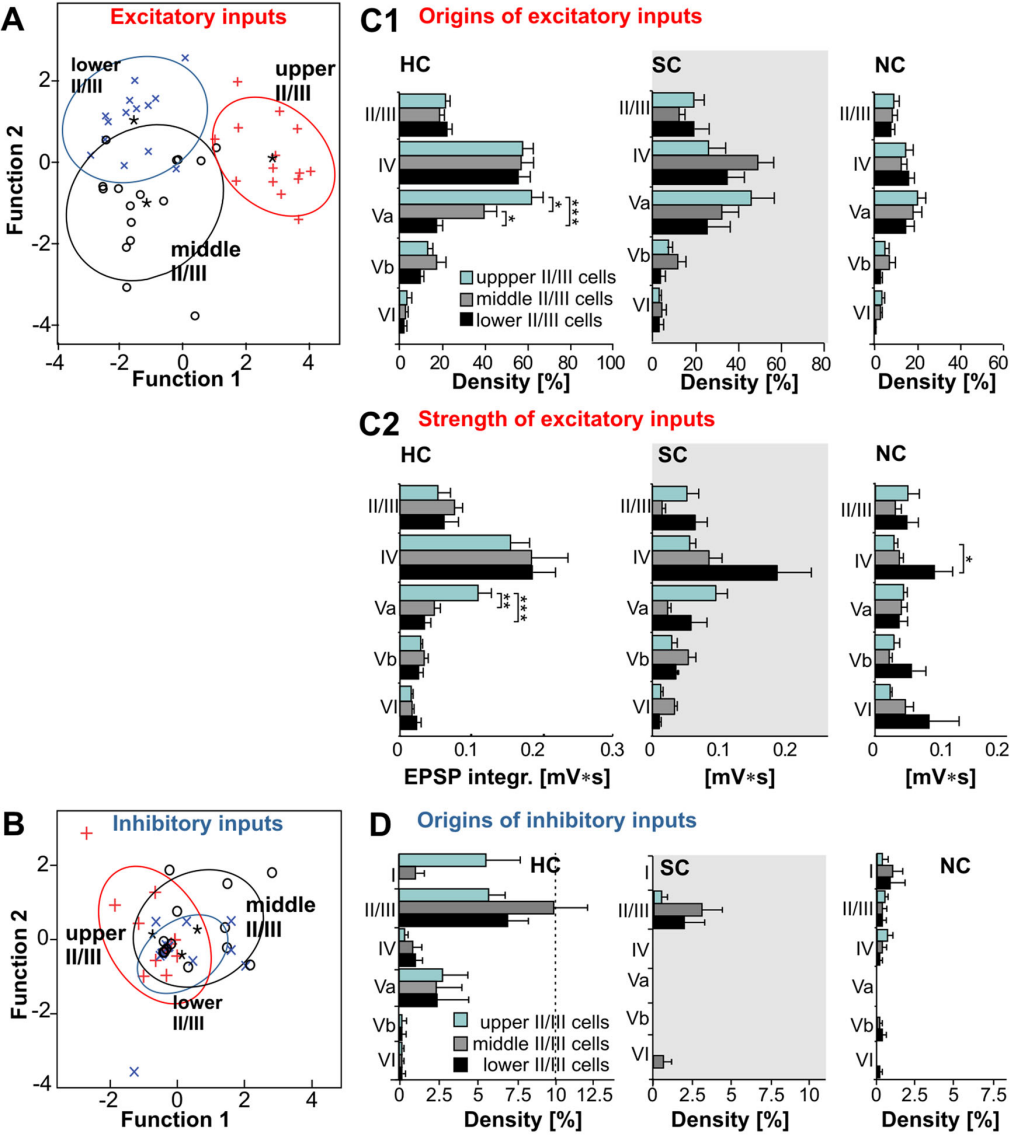
Neural populations in upper, middle and lower LII/III differ significantly in their layer-specific functional input connectivity. **a**, **b** Canonical scores plots of the properties of flash-evoked **a** excitatory and **b** inhibitory inputs (upper LII/III *n* = 14; middle LII/III *n* = 16; lower LII/III *n* = 14) following discriminant analysis of the three a priori groups. Function 1 and 2 are linear combinations of properties that best discriminate the groups. Confidence ellipses surround the group centroids (*asterisks*). **c** Layer- and column-specific density (**c1**) and strength (**c2**) of origins for excitatory inputs from the home (HC), septal (SC) and neighboring (NC) column onto pyramidal cell populations in upper, middle and lower LII/III as shown in **a**. **d** Layer- and column-specific density of origins for inhibitory inputs onto pyramidal cell populations in upper, middle and lower LII/III as shown in **b**. Data are mean ± SEM. *Asterisks* indicate significant differences between different populations. MANOVA, Bonferroni corrected: *p < 0.05, **p < 0.01, and ***p < 0.001

To visualize the representative input connectivity for the pyramidal cells in lower, middle and upper LII/III, we constructed average maps of the strength and reliability of the excitatory inputs (confidence maps, Fig. 7a) as well as of the reliability of the inhibitory inputs (Fig. 7b). Discriminant analysis based on the excitatory functional connectivity revealed that the neuronal populations of lower, middle and upper LII/III (sliding-window-derived groups) were clearly distinguishable (lower LII/III *n* = 14; middle LII/III *n* = 16; upper LII/III *n* = 14; *p* = 0.025, Wilk’s lambda, 93 % correct classification). Accordingly, the canonical scores plots showed the three groups as only weakly overlapping populations (Fig. 8a), with the most important discriminating factors being density and strength of excitatory LVa inputs from the home column. In contrast, discriminant analysis of the density of inhibitory inputs failed in reliably distinguishing groups (43 % correct classification) and the respective canonical scores plot showed extensively overlapping populations (Fig. 8b). In agreement with this, no parameter of inhibitory input connectivity revealed any significant correlation with the soma position. Consequently, whereas excitatory input properties are clearly different for pyramidal cell networks of lower, middle and upper LII/III, the inhibitory connectivity appears to be generally similar within LII/III.

### Intralaminar excitatory synaptic inputs

In general, supragranular pyramidal neurons received local excitatory inputs mainly from HC and SC fields (Fig. 2b, c). These inputs were patchy, as reflected by moderate levels of confidence in the average maps (Fig. 7b). Inputs typically consisted of one or a few EPSPs of 0.3–1.5 mV in amplitude. Within the HC, about 20 % of the stimulated fields generated excitatory inputs of weak or moderate strength (0.07 ± 0.02 mV s, *n* = 44; Fig. 8c). Even the strongest individual excitatory inputs rarely reached integral values larger than 0.15 mV s. Compared to the dominance of local synaptic inputs in other cortical layers (cf. Schubert et al. 2007), this seems comparatively weak (Holmgren et al. 2003). We investigated whether this reflected an underestimation which was possibly caused by neurons preferentially receiving inputs from their “own” local network and not from other parts of LII/III. However, we found no significant preference for the local networks, neither in density (lower LII/III, *p* = 0.09; middle LII/III, *p* = 0.5; upper LII/III *p* = 0.9) nor in the strength of excitatory inputs (lower LII/III *p* = 0.7; upper LII/III, middle LII/III, *p* = 0.7; *p* = 0.9; data not shown). For excitatory inputs originating from supragranular fields of the neighboring column, we found a distinct reduction of almost 50 % in density (*p* < 0.0001), although no significant reduction in input strength was found. The density and strength of excitatory inputs did not change significantly for supragranular fields of the septum as compared to that of the home column (density: *p* = 0.2; strength: *p* = 0.1, Fig. 8c).

### Excitatory synaptic inputs from lemniscal LIV are strong across the entire LII/III

LIV represented the most prominent source of excitatory inputs onto supragranular pyramidal neurons (Figs. 2b, c, 7a). Flash-induced inputs typically consisted of several, large amplitude EPSPs (up to 4 mV, Fig. 2b2). Thus, neurons within the home barrel provided strong and reliable excitatory inputs onto all supragranular pyramidal neurons (Fig. 7a). On average, in almost 60 % of the fields within the home barrel, photo stimulation resulted in partially very strong inputs (0.17 ± 0.03 mV s; *n* = 44, Fig. 8c).

Excitatory inputs arising from barrels in the neighboring column were generally less dense (14.2 ± 3.9 %, *p* < 0.001; *n* = 44) and weaker than those of the home barrel (0.05 ± 0.01 mV s; *p* < 0.001). However, the strength of inputs from the neighboring barrels was significantly higher for pyramidal neurons in lower LII/III (0.08 ± 0.02 mV s; *n* = 12) than for those in upper LII/III (0.03 ± 0.005 mV s; *n* = 14, *p* = 0.05). Excitatory inputs from the septa between the barrels were very heterogeneously distributed: for a number of pyramidal cells, these excitatory inputs were lacking entirely (lower LII/III 3/14, middle LII/III 3/16; upper LII/III 4/14). In the remaining neurons such inputs had densities ranging from 26 to 50 %. Whereas neurons in lower LII/III received inputs from the septum that were of similar strength as those from the barrel of the home column (0.27 ± 0.1 mV s; Fig. 8c2), those received by middle LII/III (0.08 ± 0.02 mV s) and upper LII/III (0.05 ± 0.01 mV s) were distinctively reduced. However, the differences found across all three investigated neuronal populations were not significant due to the map-to-map variability.

### Excitatory synaptic inputs from infragranular layers are dominated by “paralemniscal” LVa

The third prominent source for excitatory inputs was the infragranular LVa. Inputs arising from stimulated fields of LVa typically consisted of few EPSPs (usually 1–2, up to 5) with moderate amplitudes from 0.5 to 2 mV (Fig. 2b2). However, excitatory inputs from this layer revealed the most prominent differences between the neuronal networks in lower, middle and upper LII/III (Figs. 7a, 8c). Whereas for lower LII/III pyramidal cells LVa was typically a minor source for excitatory inputs, intracolumnar LVa was the second most dense and a highly consistent source of strong excitatory inputs onto upper LII/III pyramidal cells (61 ± 2.1 %; 0.15 ± 0.02 mV s, Figs. 7a, 8). In comparison, the intracolumnar excitatory inputs from LVa into lower and middle LII/III were significantly less dense (lower vs. upper LII/III: *p* < 0.001; lower vs. middle LII/III: *p* < 0.01; middle vs. upper LII/III: *p* < 0.01; Fig. 8c1), weaker (lower vs. upper LII/III: *p* < 0.001; middle vs. upper LII/III: *p* < 0.01; Fig. 8c2) and less consistent (Fig. 7a2). For inputs originating from LVa of the neighboring column, density and strength were generally reduced. For LVa inputs originating from below a septum, we found no significant reduction of input density and the inputs remained relatively strong networks in lower (0.10 ± 0.03 mV s) and upper LII/III (0.10 ± 0.02 mV s). Among the deeper infragranular LVb and LVI, only LVb provided a reliable source of excitation (confidence level > 80 %), typically consisting of one or a few low-amplitude EPSPs (0.3–1 mV; Fig. 7a, cf. Fig. 8).

### Inhibitory synaptic inputs

As mentioned above, our data imply similar inhibitory input patterns for all supragranular pyramidal cells, which in the home column consisted of two main sources of prominent, slowly rising and decaying hyperpolarizations at *V*_h_ = −60 mV (Fig. 2b2): (1) supragranular LII/III including LI and (2) LVa (Figs. 7b, 8d). From home column LI, some pyramidal neurons of upper (3/14 neurons) and middle LII/III (2/16) received inhibitory inputs with densities ranging from 8 to 51 % (Fig. 8d), whereas none (0/14) of the neurons in lower LII/III received such inputs. The main source was intracolumnar LII/III, providing IPSPs from on average 6–10 % of the fields (Fig. 8d). In addition, about 25 % of supragranular pyramidal cells received inhibitory inputs from LVa (Fig. 7b). These IPSPs were not prominent (Fig. 2b2) and originated from between 4 and 20 % of the fields in LVa (Fig. 8d). Occasionally, neurons received inhibitory inputs from the supragranular septal column, whereas IPSPs from the neighboring column were typically absent.

### Structural input–output relationships of pyramidal cells in LII/III

**Fig. 9:**
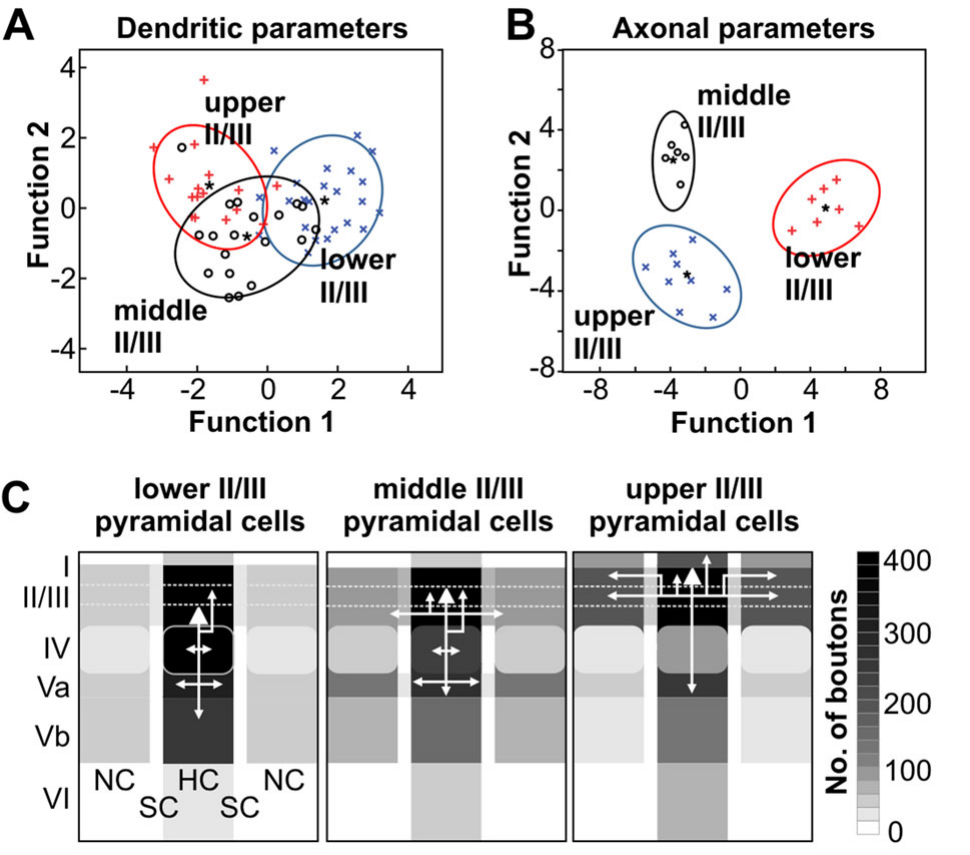
Neural populations in upper, middle and lower LII/III differ significantly in their layer-specific structural output but not input properties. **a**, **b** Canonical scores plots of (somato-)dendritic (**a** upper LII/III *n* = 20, middle LII/III *n* = 19, lower LII/III *n* = 20, *p* = 0.01, Wilk’s lambda) and axonal properties (**b** upper LII/III *n* = 9, middle LII/III *n* = 6, lower LII/III *n* = 7; *p* < 0.01). **c** Layer- and column-specific distribution of axonal boutons. Grayscale intensity represents mean numbers within each cortical area. *White arrows* mark the most prominent projections

A summary of all morphometric data is given in Tables 2 and 3. As for the functional input properties, discriminant analysis showed that the neuronal populations of lower, middle and upper LII/III were significantly different (somatodendritic parameters lower LII/III *n* = 20; middle LII/III *n* = 19, upper LII/III *n* = 20; *p* = 0.001, Wilk’s lambda, 78 % correct classification; axonal properties: lower LII/III *n* = 7; middle LII/III *n* = 6, upper LII/III *n* = 9; *p* = 0.001, 100 % correct classification). The most important discriminating properties were the morphological parameters that also showed a significant correlation with the depth of the soma. We found significant correlations with the soma position for the vertical soma diameter (*p* = 0.005), vertical and horizontal dendritic span (*p* < 0.001 each), number of primary dendrites (*p* = 0.02) and the maximal diameter of the basal dendrites (*p* = 0.01). In terms of axonal properties, we found significant correlations for bouton numbers in LI (*p* = 0.003), LIV (*p* < 0.001) and LVb (*p* = 0.007) of the home column (HC) and LI of the septal column (SC, *p* = 0.04). In agreement with the discriminant analysis, canonical scores plots displayed a partial overlap between the three groups for the dendritic properties (Fig. 9a), whereas axonal properties of the pyramidal cell populations were represented in distinct clusters (Fig. 9b).

The main distinguishing somatodendritic properties between neurons in lower, middle and upper LII/III were differences in the maximal vertical dendritic span, which was significantly longer in lower LII/III neurons (394.9 ± 76.3 μm) than for those of the upper LII/III (281.2 ± 56.5 μm, *p* = 0.002). Interestingly, the average total length of the dendrites was comparable for all groups of pyramidal cells. Evidently, a smaller vertical dendritic span was compensated by a broader horizontal span and vice versa. We found that correlations existed across all supragranular compartment values of the vertical and horizontal span of the apical dendrites (*R* = −0.44, *p* = 0.001). Thus, the appearance of atypical pyramidal cells in the upper LII/III likely reflects dendritic reorganization due to the proximity to the pial surface, since we found no differences in input maps of oblique versus straight pyramidal cells.

The axonal output connectivity showed several prominent differences between lower, middle and upper LII/III. A summary of the axonal properties is given in Tables 2 and 3; Fig. 9c. The main terminal fields of LII/III neurons were the supragranular compartment and infragranular LVa and LVb. As a whole, axonal projections of pyramidal neurons in lower LII/III were more restricted to their respective home column than those of the upper LII/III, although some horizontal or oblique collaterals projected to the neighboring septa or barrel columns (Fig. 9c, see also Fig. 4b). In comparison, pyramidal neurons in upper LII/III formed higher numbers of boutons (1) in the neighboring column (NC, lower LII/III 50 ± 25; upper LII/III 188 ± 53; *p* = 0.05, MANOVA, Bonferroni corrected; Fig. 9c) and (2) in LI of the home column (lower LII/III 41 ± 13, upper LII/III 183 ± 40, *p* < 0.01). Furthermore, for neurons of lower LII/III, large numbers of boutons were found in LIV of the home column, which is in surprising contrast to neurons in upper LII/III (lower LII/IIII 392 ± 43; upper LII/III 95 ± 20; *p* < 0.001, Fig. 9c; Table 3). After passing lower LII/III and LIV, a further prominent termination field for neurons throughout LII/III was established in LV within the home column, from which horizontal collaterals also extended into adjacent septa and neighboring columns. This transcolumnar output was prominent in LVa but also extended into LVb. LVI received only very few projections from the entire supragranular compartment. Overall, pyramidal cells in middle LII/III showed axonal projection patterns with features that were intermediate to those in lower and upper LII/III (Figs. 4, 9c).

## Discussion

**Fig. 10:**
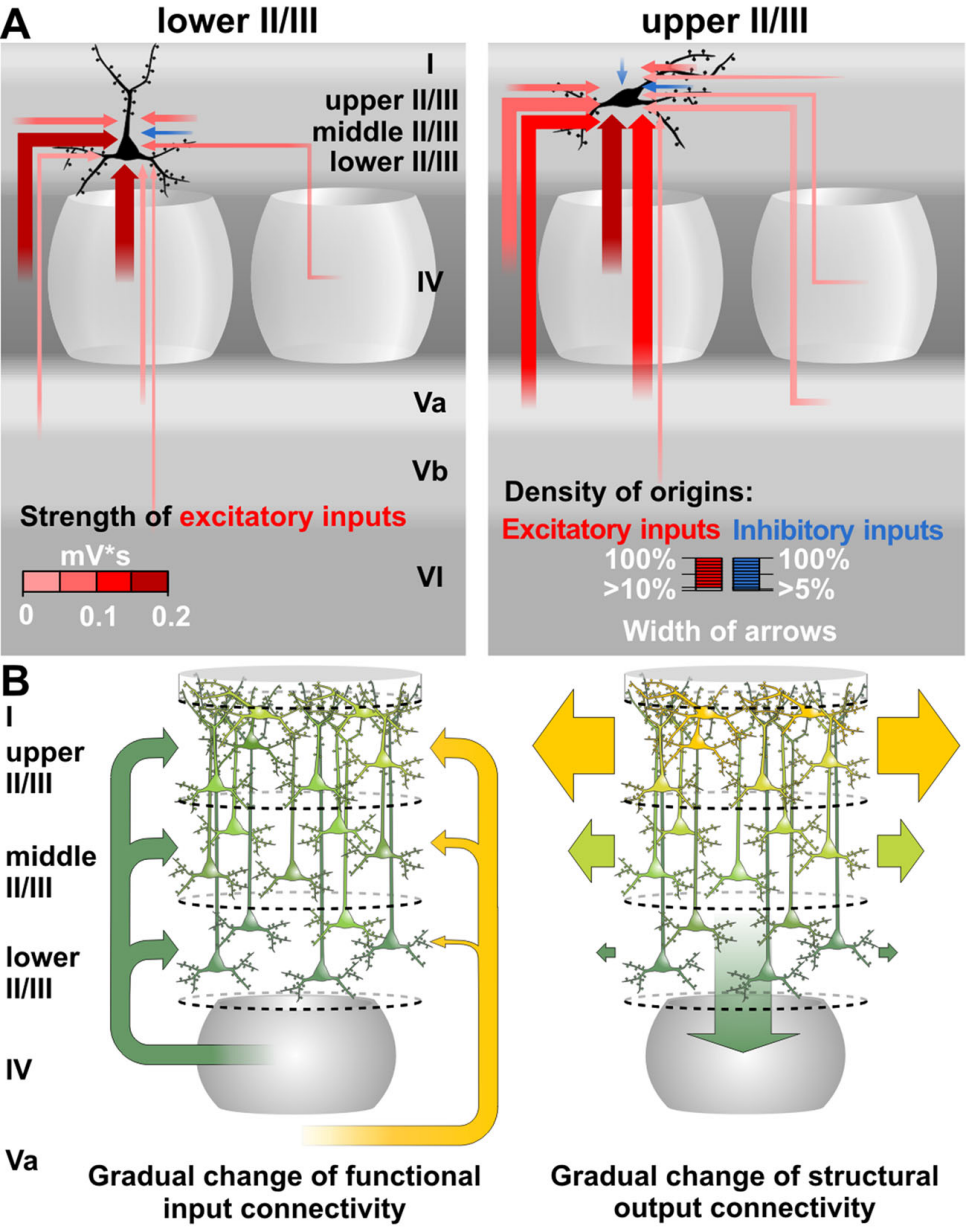
Properties of supragranular pyramidal neurons imply a structural and functional continuum. **a** Schematic drawing of the functional input connectivity of pyramidal neurons in upper and lower LII/III on neuron population (network) level. The thickness of arrows represents the layer-specific density of origins for excitatory (*red*) and inhibitory (*blue*) inputs, the color intensity average strength of excitatory inputs. For simplification, densities <10 % for excitatory and <5 % for inhibitory inputs are not shown. The position of the *arrowheads* indicates the target layer and does not imply subcellular target specificity. **b** Schematic representation of gradually changing properties of individual pyramidal neurons in LII/III*. Left panel* functional input connectivity: Neurons receiving mainly excitatory inputs from LIV (“lemniscal layer”) are given in *green*. With increasing additional excitatory inputs from LVa (“paralemniscal layer”) neurons are represented in more *yellowish* colors. Note that neurons that receive sparse or prominent inputs from LVa can be found throughout LII/III with decreasing frequency of occurrence from upper to lower LII/III, respectively. *Right panel* structural output connectivity: Gradual change of axonal projection properties within the supragranular compartment: the more *yellow* the shading of the neurons and *arrows*, the higher the preference for horizontal projections, the more *green* the shading the higher the preference for vertical projections into LIV

We present evidence that multiple circuits are embedded in the classical layer II/III of rat barrel cortex. We find structurally and functionally distinct pyramidal neurons, which display a depth-dependent gradual change of connection properties (Fig. 10). In rats, pyramidal neurons of lower LII/III are dominated in their input connectivity by the lemniscal pathway and also feedback signals to the layer of origin of their main input, which is LIV. Pyramidal cells in upper LII/III are also profoundly influenced by these lemniscal projections, but in addition can effectively interface the paralemniscal pathway by virtue of their strong inputs from LVa. The middle LII/III is, however, occupied by an intermediate population of pyramidal cells, which structurally and functionally reflects a mixture of upper and lower LII/III properties.

A major outcome of rodent in vivo calcium imaging studies within LII/III has been the finding that the topological characteristics of sensory stimuli are preserved at the columnar population level, whereas considerable variability can be found at the individual cell level. In the visual cortex this was shown for stimulus orientation (Ohki et al. 2005) and retinotopy, (Smith and Hausser 2010), in the auditory cortex for tonotopic organization (Bandyopadhyay et al. 2010; Rothschild et al. 2010). In the somatosensory cortex, principal whisker dominance appears shifted toward adjacent whiskers for a number of supragranular excitatory neurons (Kerr et al. 2007; Sato et al. 2008). This could be based on the specific circuits in which the neurons are embedded (Sato and Svoboda 2010).

These in vivo studies have concluded that multiple circuits have to be laid out within LII/III, circuits which might depend on the different target regions of the projection neurons (Chen et al. 2013). However, the investigation of the cellular circuitry organization remains subject to technical limitations and still requires dissection of the functional and structural features in vitro. Yoshimura et al. (2005) pioneered the idea of multiple segregated circuits within the supragranular compartment of rodent visual cortex, mainly based on the finding that pairs of connected pyramidal neurons receive highly correlated excitatory inputs, both locally and from LIV, a feature which was not found in unconnected pairs. Here, we studied the putative existence of multiple parallel pathways within the supragranular compartment of the rodent barrel cortex and found several new and important features. First, there is a gradual change in the composition and information content of these circuits when moving from LIV toward the pia. Secondly, lemniscal LIV and paralemniscal LVa contribute differently to the input gradient (as compared to: Shepherd and Svoboda 2005 and Bureau et al. 2006). Thirdly and importantly, this does not only apply to the functional input but also to the axonal output connectivity.

### Diverse functional input connectivity of supragranular pyramidal cells

Our results indicate strong and effective information transmission from LIV and LVa, which both represent important cortical entry points for parallel streams of sensory information, to LII/III. Previous studies showed that pyramidal neurons of the supragranular compartment integrate sensory information obtained via the lemniscal pathway from neurons in LIV barrels (Dantzker and Callaway 2000; Petersen and Sakmann 2001; Shepherd and Svoboda 2005). In agreement with this, all our supragranular neurons received extensive and powerful excitatory input from the associated home barrel. However, in addition, we found that the deeper a pyramidal cell was located, the stronger the excitatory input was that it received from LIV of neighboring columns. This gradual change of properties suggests a functional continuum wherein more superficial neurons preferentially perform whisker-specific segregation of lemniscal sensory information, while deeper pyramidal neurons integrate sensory information (i.e., associate principal and adjacent whisker input across cortical columns). The more superficially a pyramidal cell was located, the more likely it was to also receive prominent excitatory input from LVa. Therefore, upper LII/III pyramidal neurons receive lemniscal and paralemniscal information and are likely to interface with the two signal processing pathways. In middle LII/III, we found the two functional types equally distributed. These features suggest complex signal processing capabilities in the supragranular compartment: one can hypothesize that upper LII/III segregates whisker-specific lemniscal but integrates multi-whisker paralemniscal input, whereas lower LII/III integrates the whisker-specific lemniscal input, and middle LII/III might contribute to these processing streams in a flexible manner.

To which extent do networks within the supragranular compartment interact? Previous studies have found a substantial number of connections between local excitatory and inhibitory neurons (Avermann et al. 2012; Feldmeyer et al. 2006; Fino et al. 2013; Holmgren et al. 2003). In the present study, supragranular connectivity of pyramidal cells was typically reflected by moderate excitatory inputs from home, septal and neighboring columns and scattered but reliable local inhibitory modulation. Interestingly, we did not find any preference for receiving synaptic inputs from the same depth of LII/III. This implies extensive interaction between neurons of lower, middle and upper LII/III. Furthermore, we revealed a so far unreported sparse but reliable inhibitory connection between LVa and supragranular pyramidal cells, which might play a role in fine tuning the interaction of the two signal processing pathways. This input could be mediated by Martinotti-type inhibitory interneurons (Silberberg and Markram 2007), whose in vivo function is thought to support adaptive linear encoding of sensory stimuli (Murayama et al. 2009).

### Dendritic morphology and axonal projections of supragranular pyramidal cells

Previous studies have distinguished “typical” pyramidal neurons in LIII and “atypical” ones in LII (Peters and Kara 1985; Thomson and Bannister 1998; van Brederode et al. 2000). Our quantitative analysis suggests that all LII/III pyramidal neurons possess similar dendritic length to sample a comparable amount of diverse inputs. Hence, the obliqueness of those neurons close to the pial surface is likely to be a compensatory growth mechanism, without any unique functional properties so far having been associated with that morphology (Larkman and Mason 1990; Mason and Larkman 1990).

Interestingly, the axonal arborizations showed many significant differences. First, all upper LII/III pyramidal cells issued extensive transcolumnar axonal projections, whereas only a fraction of the lower LII/III pyramidal cells did. Although the sparseness of transcolumnar projections in lower LII/III pyramidal cells could be a slice artifact (Stepanyants et al. 2009), a recent in vivo study in rat barrel cortex supports the notion that the transcolumnar projections of cells in the upper supragranular compartment are more extensive (Bruno et al. 2009). Also, in vivo data from cat visual cortex exist that present evidence for similar projection patterns of supragranular pyramidal neurons as we have reported in the present study (Stepanyants et al. 2008). Previous in vitro studies have also shown differential projection targets of supragranular pyramidal cells, however, only in a qualitative manner (Barbour and Callaway 2008; Larsen and Callaway^2006^; van Brederode et al. 2000). In the quantitative study of Lübke et al. (2003), no differences have been reported between pyramidal neurons located at different depths, probably because of a bias toward recording from the heterogeneous population of middle LII/III and thereafter pooling the results. Secondly, upper LII/III not only receives strong input from LVa, but also feeds back by projecting more densely into LVa than LVb, the latter being the preferred target of lower LII/III pyramidal cells. This is in agreement with Thomson and Bannister (1998) as well as Otsuka and Kawaguchi (2008), which both could show target cell specificity for the LII to LVa and the LIII to LVb connections. Thus, “paralemniscal layers” (upper LII/III and LVa) and “lemniscal layers” (lower LII/III and LVb) have preferential reciprocal excitatory connections, which agree well with recent paired recording data (Lefort et al. 2009). Thirdly, a novel feature discovered in our study is the relatively high number of boutons carried by lower LII/III pyramidal cells into LIV. This feedback projection was considered to be mainly targeting inhibitory interneurons (Thomson and Bannister 2003). However, our previous glutamate uncaging studies showed that excitatory LIV neurons also receive functional inputs from lower LII/III (Schubert et al. 2003). Interestingly, a similar excitatory feedback loop between LIII and LIV was recently proposed for the primary auditory cortex (Barbour and Callaway 2008), suggesting a general circuit property.

### Organization of the supragranular compartment: continuum versus layers

What causes the appearance of a continuum-like organization of neuronal properties within the supragranular compartment? On the one hand, one could speculate about a novel network organizing principle whose computational power is not yet understood but could allow a more flexible functional integration of lemniscal and paralemniscal pathways. On the other hand, a set of very “thin” layers, difficult to sample specifically, may cause the impression of a continuous change of neuronal properties. Recent data on the distribution of molecular markers show differential sometimes gradually spread-out patterns (Lein et al. 2007; Sorensen et al. 2013). To clarify what the basis for the gradually changing structural and functional variations within LII/III is, we propose that future studies perform spatially fine-grained analyses of the morphological and functional properties of molecularly defined pyramidal neurons (cf. Molnar and Cheung 2006; Sugino et al. 2006). This will lead to further refinement of our concepts of the canonical and non-canonical pathways within and between cortical columns (Douglas and Martin 2007; Feldmeyer 2012; Schubert et al. 2007; Thomson and Lamy 2007).
